# Computational Investigation of Dual Filler-Incorporated Polymer Membranes for Efficient CO_2_ and H_2_ Separation: MOF/COF/Polymer Mixed Matrix Membranes

**DOI:** 10.1021/acs.iecr.2c04500

**Published:** 2023-01-26

**Authors:** Sena Aydin, Cigdem Altintas, Ilknur Erucar, Seda Keskin

**Affiliations:** † Department of Computational Science and Engineering, Koc University, Rumelifeneri Yolu, Sariyer, 34450Istanbul, Turkey; ‡ Department of Chemical and Biological Engineering, Koc University, Rumelifeneri Yolu, Sariyer, 34450Istanbul, Turkey; § Department of Natural and Mathematical Sciences, Ozyegin University, Cekmekoy, 34794Istanbul, Turkey

## Abstract

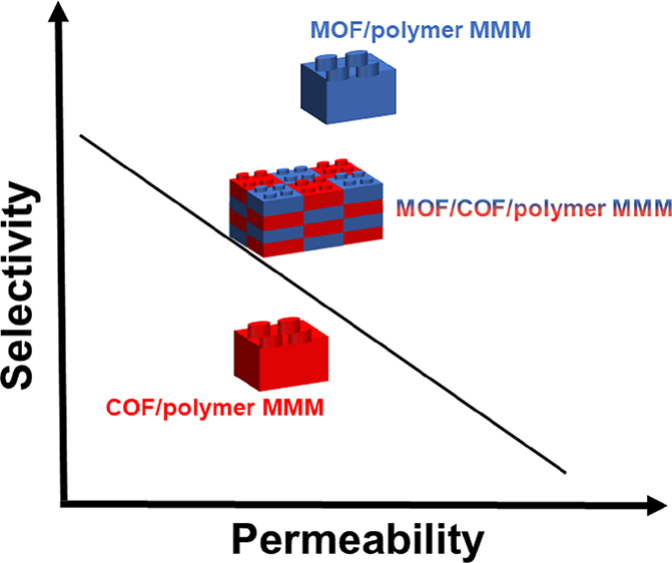

Mixed matrix membranes (MMMs) composed of two different fillers such as metal–organic frameworks (MOFs) and covalent–organic frameworks (COFs) embedded into polymers provide enhanced gas separation performance. Since it is not possible to experimentally consider all possible combinations of MOFs, COFs, and polymers, developing computational methods is urgent to identify the best performing MOF–COF pairs to be used as dual fillers in polymer membranes for target gas separations. With this motivation, we combined molecular simulations of gas adsorption and diffusion in MOFs and COFs with theoretical permeation models to calculate H_2_, N_2_, CH_4_, and CO_2_ permeabilities of almost a million types of MOF/COF/polymer MMMs. We focused on COF/polymer MMMs located below the upper bound due to their low gas selectivity for five industrially important gas separations, CO_2_/N_2_, CO_2_/CH_4_, H_2_/N_2_, H_2_/CH_4_, and H_2_/CO_2_. We further investigated whether these MMMs could exceed the upper bound when a second type of filler, a MOF, was introduced into the polymer. Many MOF/COF/polymer MMMs were found to exceed the upper bounds showing the promise of using two different fillers in polymers. Results showed that for polymers having a relatively high gas permeability (≥10^4^ barrer) but low selectivity (≤2.5) such as PTMSP, addition of the MOF as the second filler can have a dramatic effect on the final gas permeability and selectivity of the MMM. Property–performance relations were analyzed to understand how the structural and chemical properties of the fillers affect the permeability of the resulting MMMs, and MOFs having Zn, Cu, and Cd metals were found to lead to the highest increase in gas permeability of MMMs. This work highlights the significant potential of using COF and MOF fillers in MMMs to achieve better gas separation performances than MMMs with one type of filler, especially for H_2_ purification and CO_2_ capture applications.

## 1 Introduction

Membrane-based gas separation is an effective technology to capture carbon dioxide from natural gas and flue gas, to purify hydrogen and olefins from nitrogen, to dehumidify air and natural gas, and to separate different hydrocarbon pairs including alkanes/alkenes under easy-to-operate and energy-efficient conditions.^1^ Polymeric membranes suffer from a well-known gas permeability–selectivity trade-off as highlighted by Robeson;^2−4^ therefore, current membrane research is focused on the discovery of new membrane materials with enhanced gas permeability and selectivity properties to surpass the upper bound of polymeric membranes.^5−7^ Mixed matrix membranes (MMMs), which consist of a polymer incorporated with an organic/inorganic porous filler, offer enhanced gas separation performances through the combination of easy processability of polymers with high gas permeability/selectivity of fillers such as zeolites, graphene oxides, carbon molecular sieves, metal–organic frameworks (MOFs), and covalent–organic frameworks (COFs).^8−11^ These MMMs have been used for many important gas separations such as H_2_ purification and air purification.^12^

The metal nodes and organic linkers that make up MOFs are connected in a variety of topologies, providing vast space for structural tunability, with a wide range of surface areas, well-defined pore size distributions, and high porosity.^13^ COFs generally consist of B, C, O, N, and Si elements covalently linked to organic linkers.^14^ The structural and chemical diversity of MOFs is currently represented with more than 110,000 MOFs available in the Cambridge Structural Database,^15^ while the most recent COF database now includes more than 700 synthesized COFs.^16^ Both experimental and computational studies showed that many MOF and COF membranes can exhibit high gas permeabilities.^17,18^ Synthesis of thin-film membranes from crystalline materials is challenging due to many issues such as poor interactions with the substrates, crack formation, and moisture instability, which affect the practical implementation and scalability of thin-film MOF and COF membranes.^19^ Therefore, MOFs and COFs have been recently used as filler particles in polymers to make MMMs.^20^ Incorporation of MOFs as fillers into polymers has led to many MOF/polymer MMMs, which offer higher gas permeability and/or selectivity than the pure polymer membrane, but these MMMs generally suffer from compatibility issues between MOFs and polymers due to the inorganic nature of MOFs.^21^ On the other hand, COFs offer better interfacial compatibility with polymers compared to MOFs thanks to the completely organic structure of COFs.^21^ COFs generally have larger pore sizes than MOFs, which offer high gas permeability but limited selectivity since both gas molecules can pass through these large pores.^22^

Generating MMMs having both high gas permeability and high selectivity can be possible using dual types of fillers via combining two types of porous materials with a polymer. In the first experimental report on the dual filler-incorporated MMMs, Zornoza et al.^23^ examined MMMs where a MOF (HKUST-1 or ZIF-8) and a zeolite (S1C) were incorporated into polysulfone (PSF) to generate HKUST-1/S1C/PSF and ZIF-8/S1C/PSF MMMs and tested them for separation of equimolar CO_2_/CH_4_, CO_2_/N_2_, O_2_/N_2_, and H_2_/CH_4_ mixtures. The highest CO_2_/CH_4_ and CO_2_/N_2_ selectivities (22.4 and 38, respectively) were attained with the HKUST-1/S1C/PSF MMM, whereas selectivities of single filler-incorporated MMMs, HKUST-1/PSF MMM (15.8 and 22.9, respectively) and ZIF-8/PSF MMM (19.3 and 19.8, respectively), were found to be lower at 2.75 bar and 308 K. HKUST-1/S1C/PSF MMMs showed higher O_2_ permeabilities and O_2_/N_2_ selectivities (1.7 Barrer and 8.1, respectively) than the single filler-incorporated HKUST-1/PSF MMM (2.2 Barrer and 5.1, respectively).

Two different MOFs, MIL-101(Cr) and ZIF-8, were incorporated into PSF and CO_2_/CH_4_ separation performances of MMMs were tested at 2 bar and 308 K.^24^ The selectivity of MIL-101(Cr)/ZIF-8/PSF MMM (40) was reported to be higher than the selectivities of MMMs having a single filler, MIL-101(Cr)/PSF MMM (24) and ZIF-8/PSF MMM (22), and the neat polymer (23). In addition to the high CO_2_/CH_4_ selectivity, CO_2_ permeability of MIL-101(Cr)/ZIF-8/PSF MMM (14.2 Barrer) was higher than the CO_2_ permeabilities of the MIL-101(Cr)/PSF MMM (8.9 Barrer), ZIF-8/PSF MMM (13.7 Barrer), and PSF membrane (4.7 Barrer). CuBDC and ZIF-8 were incorporated into the pure ODTA-TMPDA polymer to study the separation of an equimolar CO_2_/CH_4_ mixture at 1 bar and 298 K.^25^ ZIF-8/CuBDC/ODTA-TMPDA MMMs with different ZIF-8 and CuBDC filler loadings showed improved CO_2_ permeability compared to the pure ODTA-TMPDA membrane, and the highest CO_2_/CH_4_ selectivity (47) was obtained with the ZIF-8/CuBDC/ODTA-TMPDA MMM compared to the selectivities of the ODTA-TMPDA, ZIF-8/ODTA-TMPDA MMM, and CuBDC/ODTA-TMPDA MMM (36, 37, and 43 respectively).

As this literature summary suggests, there is a significant potential in utilizing dual types of fillers in MMMs to achieve higher gas permeabilities and selectivities compared to those offered by MMMs having a single type of filler. Examining gas separation performances of MMMs having various combinations of MOFs and COFs as dual fillers is required to unlock the full potential of MOF/COF/polymer MMMs for different gas separations. In this work, we aimed to predict gas separation performances of MOF/COF/polymer MMMs and to identify the best performing MOF/COF/polymer MMMs for H_2_ and CO_2_ separations. Considering the very large number of existing MOFs, COFs, and polymers, it is not possible to experimentally test every single combination of MOF/COF/polymer MMMs for a target gas separation. Motivated by this, we introduced a computational approach based on molecular simulations and theoretical gas permeation models to predict gas permeabilities and selectivities of almost a million different types of MOF/COF/polymer MMMs. We studied 966,330 MMMs obtained from the combination of 1193 MOFs, 589 COFs, and 4 different polymers, which represent the largest number and variety of MOF/COF/polymer MMMs examined to date. Permeabilities and selectivities of these MOF/COF/polymer MMMs have been studied for 5 important gas separations: H_2_/N_2_, H_2_/CH_4_, H_2_/CO_2_, CO_2_/N_2_, and CO_2_/CH_4_. Using the very large amount of membrane performance data produced by molecular simulations and permeation models, we analyzed structure–performance relations to understand how the structural properties of MOFs and COFs affect the permeability and selectivity of the resulting MOF/COF/polymer MMMs. Results of this work will provide significant insights into identifying the most useful MOF/COF pairs that can be utilized as dual fillers in polymer membranes and accelerate the design and development of the ideal MOF/COF/polymer MMMs for H_2_ purification and CO_2_ capture applications.

## 2 Computational Methodologies

**Scheme 1:**
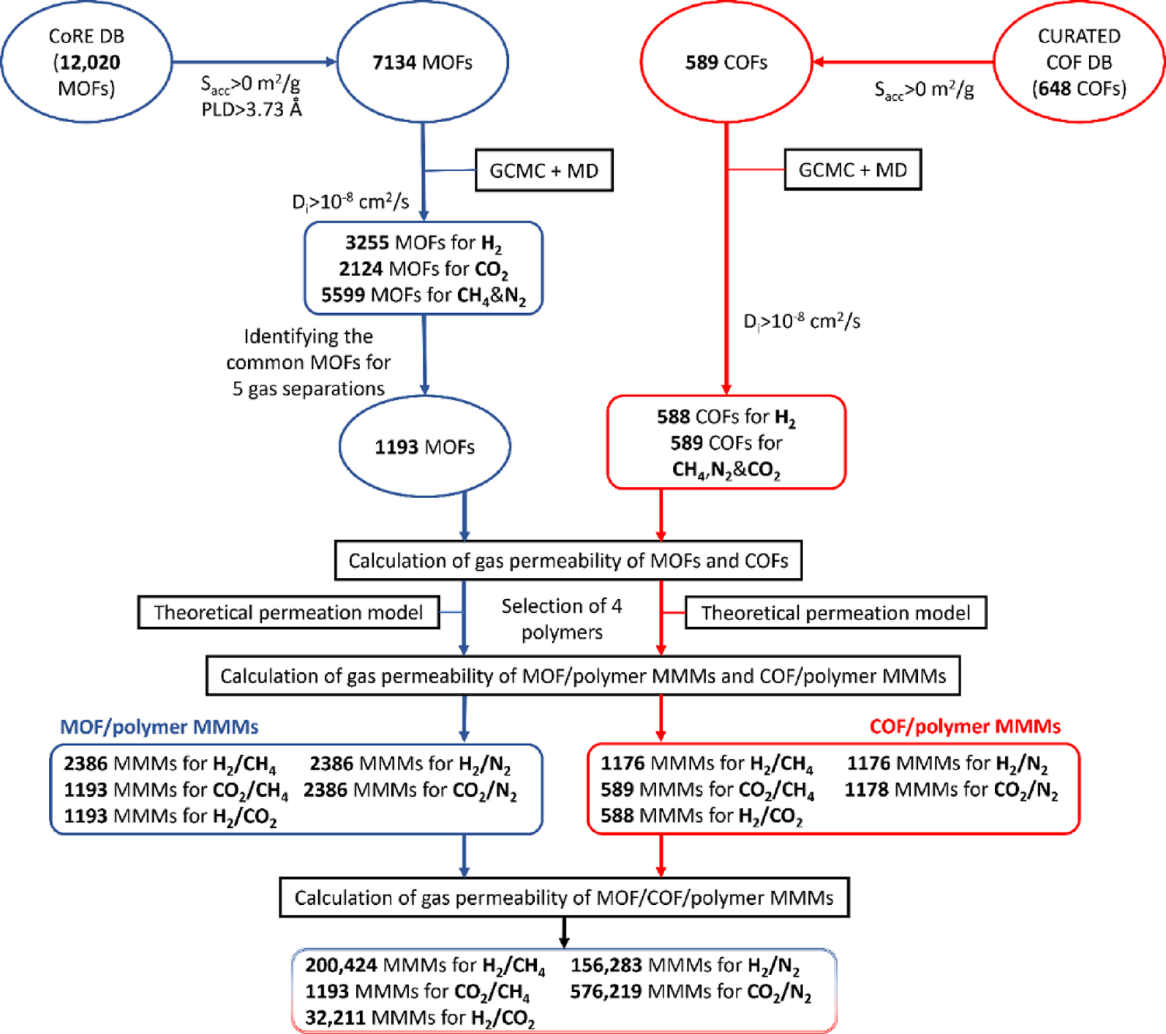
Computational Approach of This Work Combining Molecular Simulations of MOFs and COFs with Theoretical Permeation Models to Predict H_2_, N_2_, CH_4_, and CO_2_ Permeabilities of MOF/COF/Polymer MMMs

We designed a computational approach combining molecular simulations of gas adsorption and diffusion in MOFs and COFs together with the theoretical permeation models to calculate H_2_, N_2_, CH_4_, and CO_2_ permeabilities of MOF/COF/polymer MMMs as shown in Scheme 1. We initially considered 12,020 MOFs taken from the most recent computation-ready, experimental (CoRE) MOF database^26^ and 648 COFs taken from the Clean, Uniform, and Refined with Automatic Tracking from Experimental Database (CURATED)^16^ as fillers for MMMs. Structural properties of MOFs and COFs such as the largest cavity diameter (LCD), pore limiting diameter (PLD), accessible surface area (*S*_acc_), and porosity were computed via Zeo++ software.^27^ We used a probe radius of 1.86 Å representing the radius of N_2_ to predict *S*_acc_ of MOFs and COFs and only focused on materials with PLD > 3.73 Å and *S*_acc_ > 0 m^2^/g to be able to compute gas permeability. Adsorption amounts of H_2_, N_2_, CH_4_, and CO_2_ (H_2_, N_2_, and CH_4_) in MOFs (COFs) were calculated using grand canonical Monte Carlo (GCMC) simulations at 1 bar and 298 K, and molecular dynamics (MD) simulations were performed at the adsorbed gas loadings to compute self-diffusivities of gases in MOFs and COFs as described in detail in our previous studies.^22,28^ To obtain statistically accurate data from MD simulations, we discarded the MOFs and COFs for which self-diffusion coefficients of gases were computed to be less than 10^–8^ cm^2^/s. After this refinement, we ended up with 2124, 3255, 5599, and 5599 MOFs for which CO_2_, H_2_, CH_4_, and N_2_ adsorption and diffusion data were generated by molecular simulations.^28^ Since we aimed to study five different gas separations, H_2_/N_2_, H_2_/CH_4_, H_2_/CO_2_, CO_2_/N_2_, and CO_2_/CH_4_, we focused on the 1193 common MOFs for which these data are available for all four gases.

We previously reported H_2_ (N_2_ and CH_4_) adsorption and diffusion data at 1 bar and 298 K for 588 (589) COFs using molecular simulations.^22^ Additionally, we performed GCMC and MD simulations for CO_2_ in this work using RASPA software (version 2.0.35)^29^ and obtained CO_2_ uptakes and self-diffusion coefficients in COFs at 1 bar and 298 K. Lennard-Jones 12-6 (LJ) and Coulomb potentials were used to describe dispersion and electrostatic interactions, respectively. CO_2_ was modeled as a linear and three-site rigid molecule, and electrostatic interactions between COF and CO_2_ molecules were considered.^30^ The LJ parameters of atoms in COFs were acquired from the universal force field (UFF),^31^ and these atoms were already assigned with the density-derived electrostatic and chemical (DDEC) charges.^16^ The details of simulation parameters and conditions can be found in Table S1.

After adsorption and diffusion data of gases in MOFs and COFs were obtained from GCMC and MD simulations, permeabilities of MOFs and COFs for gas species *i* (*P*_*i*_^MOF^ and *P*_*i*_^COF^) were calculated as shown in eqs 1 and 2 using the adsorbed concentration of gas *i* (*c_i_*), the self-diffusion coefficient of gas *i* (*D_i_*), and the feed side pressure of the membrane (*f*) at 1 bar. The permeate side of the membrane was assumed to be at vacuum:^32^
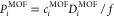
1
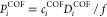
2

We then used a theoretical permeation model, the Maxwell model,^33,34^ to compute the gas permeabilities of MMMs having a single type of filler. All permeability data were obtained for 1 bar and 298 K. Permeabilities of MOF/polymer MMMs (*P*_*i*_^MOF/polymer MMM^) and COF/polymer MMMs (*P*_*i*_^COF/polymer MMM^) were calculated as follows:
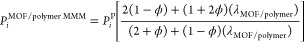
3
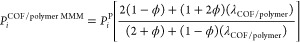
4in which *P*_*i*_^P^ is the gas permeability of the neat polymer for gas species *i* that we obtained from the literature (listed in Table S2), λ_MOF/polymer_ = *P*_*i*_^MOF^/*P*_*i*_^P^, λ_COF/polymer_ = *P*_*i*_^COF^/*P*_*i*_^P^, and ϕ describes the volume fraction of the filler (either the MOF or COF) in the polymer matrix. The Maxwell model is one of the most commonly used theoretical permeation models and known to be valid at low filler loadings (ϕ ≤ 0.2).^33^ Thus, the volume fraction of the filler was set to 0.2. Selectivities of MOF/polymer MMMs and COF/polymer MMMs for gas species *i* over *j* were computed as the ratio of the corresponding gas permeabilities as follows:
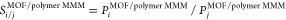
5
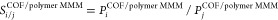
6

We specifically focused on 4 different polymers, PTMSP, PTMSP-co(95/5), Teflon AF-2400, and PIM-1^35−37^ for 5 different gas separations studied in this work. We selected PTMSP, PTMSP-co(95/5), Teflon AF-2400, and PIM-1 among the polymers which define the Robeson’s upper bound for each gas separation. PTMSP and PIM-1 were studied for CO_2_/N_2_ separation since PTMSP has a high CO_2_ permeability and PIM-1 offers a relatively high CO_2_/N_2_ selectivity. For CO_2_/CH_4_, we focused on a highly selective polymer PIM-1. For H_2_/N_2_, H_2_/CH_4_, and H_2_/CO_2_ separations, we studied PTMSP-co(95/5) since it has a high H_2_ permeability. Teflon AF-2400 and PIM-1 were studied for H_2_/CH_4_ and H_2_/N_2_ separations due to their relatively high selectivity. Experimental gas permeabilities and selectivities of these polymers were collected from the literature and are listed in Table S2. As a result, we focused on 2386 MOF/polymer MMMs and 589 COF/polymer MMMs for CO_2_/N_2_ separation, 1193 MOF/polymer MMMs and 589 COF/polymer MMMs for CO_2_/CH_4_ separation, 2386 MOF/polymer MMMs and 1176 COF/polymer MMMs for H_2_/CH_4_ separation, 2386 MOF/polymer MMMs and 1176 COF/polymer MMMs for H_2_/N_2_ separation, and 1193 MOF/polymer MMMs and 1176 COF/polymer MMMs for H_2_/CO_2_ separation.

After calculating the gas permeability and selectivity of MOF/polymer MMMs and COF/polymer MMMs, we identified 1193 common MOF/polymer MMMs and 589 COF/polymer MMMs for H_2_/N_2_, H_2_/CH_4_, CO_2_/N_2_, CO_2_/CH_4_, and H_2_/CO_2_ separations. To compute the gas permeability of MOF/COF/polymer MMMs for gas species *i* (*P*_*i*_^MOF/COF/polymer MMM^), we used an approximate model, which was previously defined to express the permeability in binary blends of polymers and copolymers:^38^
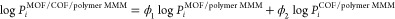
7

Here, ϕ_1_ and ϕ_2_ represent the filler volume fractions of the MOF/polymer MMM and the COF/polymer MMM in the MOF/COF/polymer MMM. They were used as 0.5 to assume that equal volumes of MOF/polymer and COF/polymer MMMs are utilized to obtain the MOF/COF/polymer MMM. The validity of this approximate model was previously shown by comparing its predictions for CO_2_, N_2_, and CH_4_ permeabilities of the ZIF-8/S1C/PSF MMM and CuBTC/S1C/PSF MMM with the experimental measurements.^39^ In this work, we also calculated H_2_, N_2_, O_2_, CO_2_, and CH_4_ permeabilities of MOF/S1C/PSF MMMs using molecular simulations and eq 7 for two different MOFs (ZIF-8 and CuBTC) at the same conditions with the experiments reported in the literature. Gas permeabilities of two MOFs were calculated in this work, while data for S1C and PSF were taken from the literature.^23^Figure S1 shows that there is good agreement between the calculated and experimental permeabilities of ZIF-8/S1C/PSF MMMs and HKUST/S1C/PSF MMMs for H_2_, N_2_, O_2_, CO_2_, and CH_4_, demonstrating the validity of our approach for assessing separation performances of dual filler-incorporated MMMs.

It was recently shown that MOFs generally have higher selectivities compared to COFs in membrane-based gas separation applications.^22^ Thus, we specifically focused on COF/polymer MMMs located below the upper bound due to their low selectivity for 5 different gas separations in this work and explored if they can exceed the upper bound when a second type of filler, a MOF, is introduced into the polymer. The numbers of all COF/polymer MMMs located below the upper bound and used to generate MOF/COF/polymer MMMs for each separation are given in Table S3. To quantify the improvements of using dual fillers (a MOF and a COF) instead of using only a COF filler in MMMs, we defined the percent (%) changes in gas permeabilities and selectivities of MOF/COF/polymer MMMs, which are located above the upper bound with respect to the gas separation performances of COF/polymer MMMs. The percent changes in gas permeability and selectivity were calculated as follows:
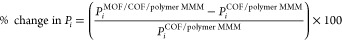
8
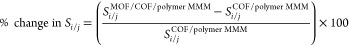
9

Overall, we calculated the gas permeabilities and selectivities of 576,219 MOF/COF/polymer MMMs for CO_2_/N_2_, 1193 MOF/COF/polymer MMMs for CO_2_/CH_4_, 200,424 MOF/COF/polymer MMMs for H_2_/CH_4_, 156,283 MOF/COF/polymer MMMs for H_2_/N_2_, and 32,211 MOF/COF/polymer MMMs for H_2_/CO_2_ separations.

## 3 Results and Discussion

After computing the permeability and selectivity of dual filler-incorporated MMMs, we examined their locations with respect to the upper bound for each gas separation as we will discuss in detail below. Many MOF/COF/polymer MMMs were found to exceed the upper bound. The number of MOF/COF/polymer MMMs that can exceed the upper bound for each gas separation of interest is given in Table 1 together with the corresponding number of COF/polymer MMMs and MOF/polymer MMMs used for their modeling. As an example for reading the table, 120 COF/PTMSP-co(95/5) MMMs were found to be below the upper bound for H_2_/N_2_ separation. These 120 COF/PTMSP-co(95/5) MMMs and 1193 MOF/PTMSP-co(95/5) MMMs were combined to generate 143,160 MOF/COF/PTMSP-co(95/5) MMMs. 41,603 MMMs exceeded the upper bound, and they were generated from combinations of 115 COF/PTMSP-co(95/5) MMMs and 652 MOF/PTMSP-co(95/5) MMMs.

**Table 1:** The Numbers of MOF/COF/Polymer MMMs That Exceed the Upper Bound and the Numbers of MOF/Polymer and COF/Polymer MMMs Used to Generate Them

polymer	MOF/COF/polymer MMM	COF/polymer MMM	MOF/polymer MMM
CO_2_/N_2_			
PTMSP	119,622	375	652
PIM-1	2020	2	1104
CO_2_/CH_4_			
PIM-1	1143	1	1143
H_2_/CH_4_			
PTMSP-co(95/5)	40,904	158	574
Teflon AF-2400	7830	8	1143
H_2_/N_2_			
PTMSP-co(95/5)	41,603	115	653
PIM-1	9735	9	1131
H_2_/CO_2_			
PTMSP-co(95/5)	22,090	27	1018

### 3.1 CO_2_/N_2_ Separation

**Figure 1:**
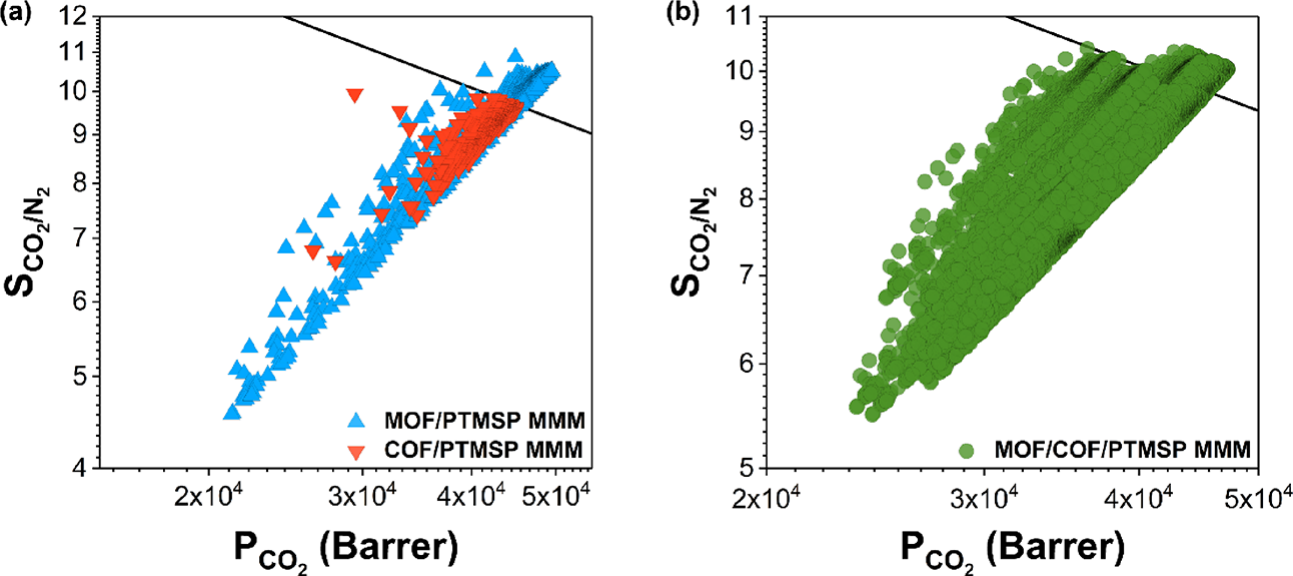
Calculated CO_2_ permeabilities and CO_2_/N_2_ selectivities of (a) 480 COF/PTMSP MMMs and 1193 MOF/PTMSP MMMs and (b) 572,640 MOF/COF/PTMSP MMMs generated from them. Robeson’s upper bound is shown with the black line.

We calculated CO_2_ permeabilities and CO_2_/N_2_ selectivities of 480 COF/PTMSP MMMs, which were all located below the upper bound (red data), and 1193 MOF/PTMSP MMMs (blue data) as shown in Figure 1a. MOF/COF/PTMSP MMMs (572,640) were generated from them, and their CO_2_ permeabilities and CO_2_/N_2_ selectivities were computed. A total of 119,622 MMMs out of 572,640 MOF/COF/polymer MMMs exceeded the Robeson’s upper bound as shown in Figure 1b. Throughout this work, we specifically focused on the MOF/COF/polymer MMMs that exceed the upper bound and compared their gas permeability and selectivity with those of COF/polymer MMMs that were below the upper bound to understand how incorporation of a second type of filler, a MOF, can improve the gas separation performance of a COF-incorporated polymer membrane.

**Figure 2:**
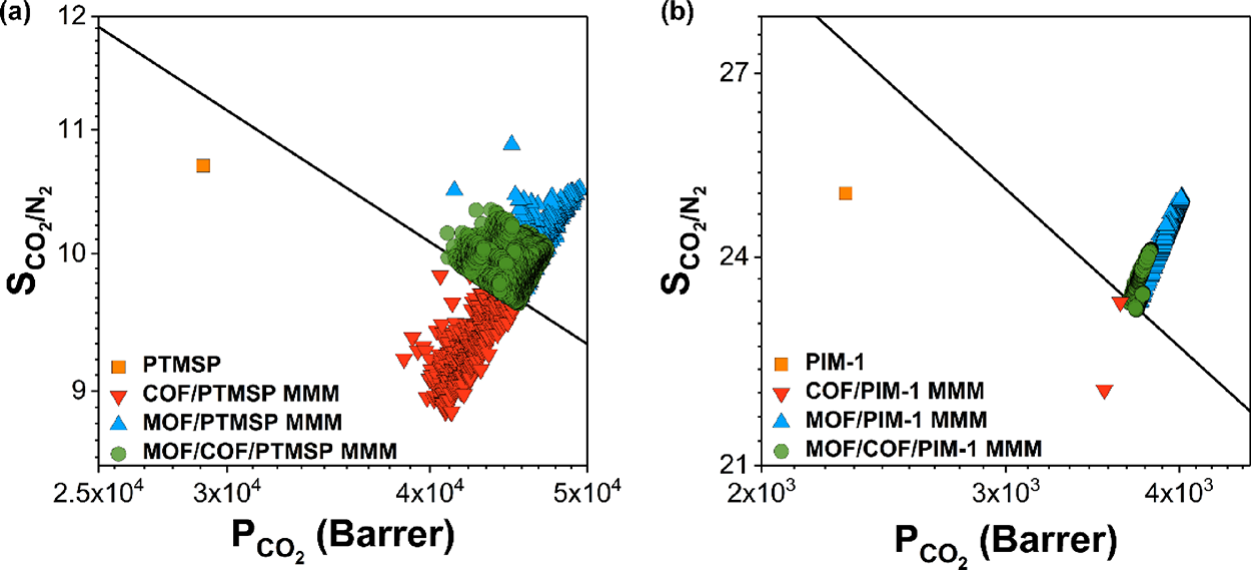
Calculated CO_2_ permeabilities and CO_2_/N_2_ selectivities of (a) 652 MOF/PTMSP MMMs, 375 COF/PTMSP MMMs, and 119,622 MOF/COF/PTMSP MMMs generated from them and (b) 1104 MOF/PIM-1 MMMs, 2 COF/PIM-1 MMMs, and 2020 MOF/COF/PIM-1 MMMs generated from them. Robeson’s upper bound is shown with the black line.

CO_2_ permeabilities and CO_2_/N_2_ selectivities of MOF/COF/polymer MMMs that exceed the upper bound are shown in Figure 2 together with permeabilities and selectivities of MOF/polymer MMMs and COF/polymer MMMs that were used to generate them. We considered two different polymers, PTMSP and PIM-1, as the matrix of MMMs for CO_2_/N_2_ separation. PTMSP has a very high CO_2_ permeability and mediocre CO_2_/N_2_ selectivity (29,000 Barrer and 10.7, respectively), whereas PIM-1 has a relatively high CO_2_/N_2_ selectivity (25) with a CO_2_ permeability of 2300 Barrer. We examined how gas separation performances of these two polymers are changed by the addition of dual fillers, MOFs and COFs.

Figure 2a shows CO_2_ permeabilities and CO_2_/N_2_ selectivities of 119,622 MOF/COF/PTMSP MMMs (green data) generated from the combinations of 375 COF/PTMSP MMMs (red data) and 652 MOF/PTMSP MMMs (blue data). CO_2_ permeabilities of MOF/PTMSP MMMs were computed to be generally higher, 4.14 × 10^4^–4.95 × 10^4^ Barrer, than those of COF/PTMSP MMMs, 3.86 × 10^4^–4.50 × 10^4^ Barrer. Similarly, CO_2_/N_2_ selectivities of MOF/PTMSP MMMs were calculated to be higher (9.6–10.9) than those of COF/PTMSP MMMs (8.9–9.8). Thus, the resulting MOF/COF/PTMSP MMMs have a higher CO_2_ permeability, 4.10 × 10^4^–4.72 × 10^4^ Barrer, and a higher CO_2_/N_2_ selectivity, 9.6–10.3, compared to COF/PTMSP MMMs. Figure 2b shows that out of 3579 MOF/COF/PIM-1 MMMs, 2020 MOF/COF/PIM-1 MMMs (green data) generated from 2 COF/PIM-1 MMMs (red data) and 1104 MOF/PIM-1 MMMs (blue data) exceed the upper bound. CO_2_ permeabilities and CO_2_/N_2_ selectivities of these 2020 MOF/COF/polymer MMMs were computed to vary between 3.68 × 10^3^ and 3.82 × 10^3^ Barrer and 23.2 and 24.1, respectively, while CO_2_ permeabilities and CO_2_/N_2_ selectivities of COF/PIM-1 MMMs are in between 3.53 × 10^3^ and 3.63 × 10^3^ Barrer and 22.0 and 23.3, respectively.

**Figure 3:**
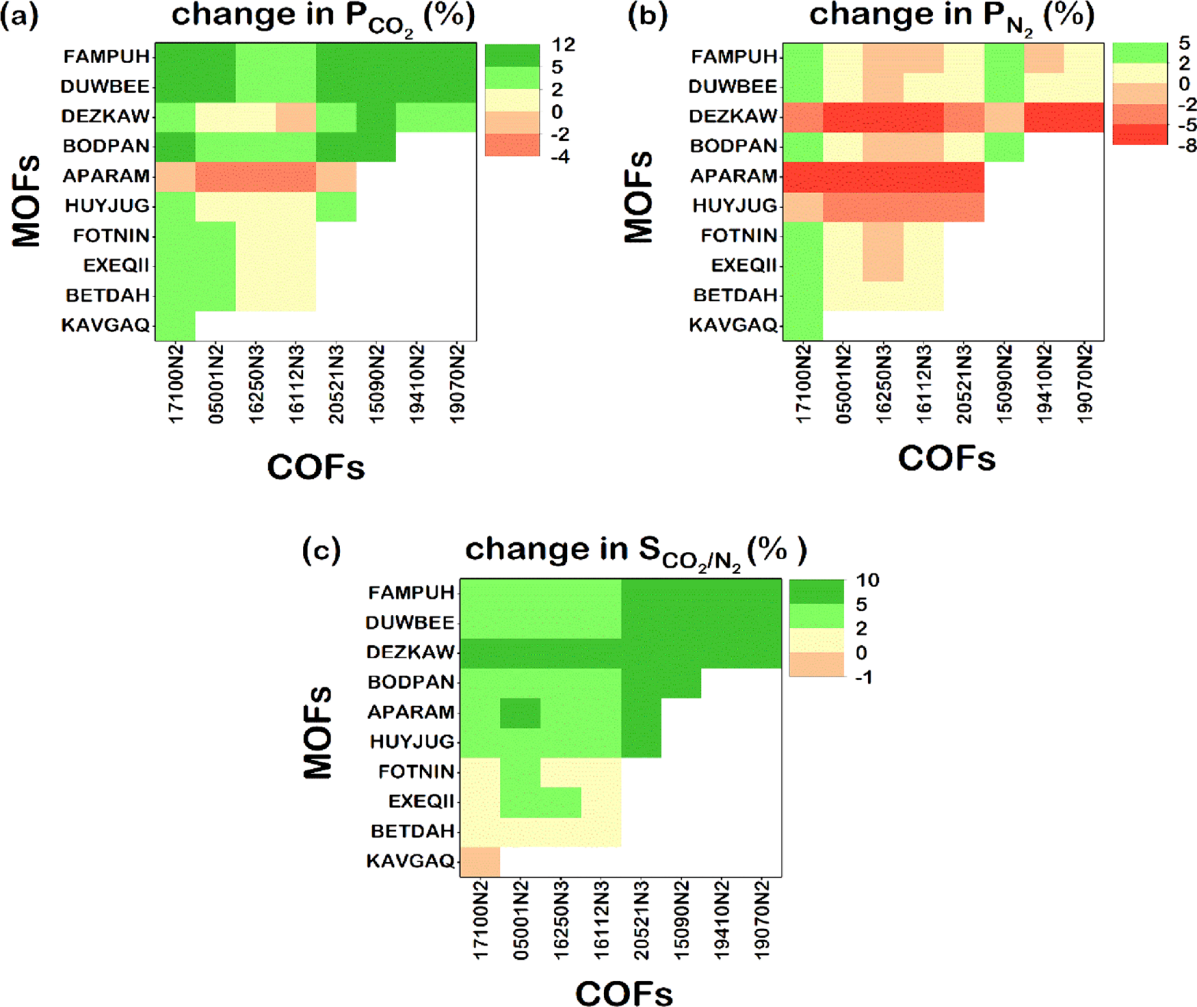
Calculated percent changes in (a) CO_2_ permeabilities, (b) N_2_ permeabilities, and (c) CO_2_/N_2_ selectivities of representative MOF/COF/PTMSP MMMs with respect to COF/PTMSP MMMs for the selected set of MOF–COF pairs.

These results show the high potential of improving gas separation performance of only COF-incorporated MMMs by the addition of the MOFs as the second filler type. To better understand the results, we focused on MOF/COF/PTMSP MMMs, which overcome the upper bound by showing (i) an increase both in CO_2_ permeability and CO_2_/N_2_ selectivity, (ii) an increase both in CO_2_ and N_2_ permeabilities without any change in CO_2_/N_2_ selectivity, and (iii) an increase in CO_2_/N_2_ selectivity especially due to the decrease in N_2_ permeability compared to separation performances of COF/PTMSP MMMs. To quantify how incorporation of a second type of filler, a MOF, can improve the gas separation performance of a COF-incorporated polymer membrane, we calculated the percent changes in CO_2_ permeabilities, N_2_ permeabilities, and CO_2_/N_2_ selectivities of 119,622 MOF/COF/PTMSP MMMs with respect to 375 COF/PTMSP MMMs. Figure S2 shows that when MOF and COF fillers are used together in PTMSP instead of only a COF filler, percent changes in *P*_CO_2__, *P*_N_2__, and *S*_CO_2_/N_2__ varied between −4 and 13%, −9 and 7%, and −1 and 11%, respectively. We presented these percent changes for a representative set of MOF/COF/PTMSP MMMs in Figure 3a–c where *x* and *y* axes illustrate the identity of COFs and MOFs in COF/PTMSP MMMs and MOF/PTMSP MMMs used for generating MOF/COF/PTMSP MMMs. If there is a more than 2% increase in the permeability or selectivity of an MOF/COF/PTMSP MMM compared to that of a corresponding COF/PTMSP MMM, the MOF–COF matching is shown with green. If using dual fillers instead of using only a COF filler in MMMs causes a small change of 0–2% (a decrease) in the permeability or selectivity, the MOF–COF matching is shown with yellow (red). If the resulting MOF/COF/polymer MMM still remains below the upper bound, it is shown by white. For example, when the MOF, DUWBEE, and the COF, 15090N2, were used together in PTMSP, the resulting MMM surpasses the upper bound thanks to an 11.7% (5.3%) increase in CO_2_ (N_2_) permeability and a 6.1% increase in selectivity compared to the only COF-incorporated polymer membrane, the 15090N2/PTMSP MMM. Here, the MOF, DUWBEE, offers a very high CO_2_ permeability (1.28 × 10^6^ Barrer), which can be attributed to its high CO_2_ uptake (2.19 mol/kg) and fast CO_2_ diffusion (2.02 × 10^–4^ cm^2^/s). On the other hand, the COF, 15090N2, is much less CO_2_-permeable (1.27 × 10^5^ Barrer) due to its lower CO_2_ adsorption (0.98 mol/kg) and slower diffusivity (7.99 × 10^–5^ cm^2^/s). A MOF/COF/polymer MMM example with improved selectivity is the HUYJUG/20521N3/PTMSP MMM where incorporation of HUYJUG and 20521N3 into PTMSP resulted in a membrane with a 2.9% higher CO_2_ permeability, a 2.8% lower N_2_ permeability, and a 5.8% higher selectivity compared to the only COF-incorporated membrane. The HUYJUG/20521N3/PTMSP MMM overcomes the upper bound by a CO_2_ permeability of 4.39 × 10^4^ Barrer and a CO_2_/N_2_ selectivity of 9.9. Another interesting example is the FOTNIN/17100N2/PTMSP MMM, which overcomes the upper bound due to a ∼4% increase in CO_2_ and N_2_ permeabilities without a significant change in CO_2_/N_2_ selectivity. The increase in gas permeabilities is attributed to higher CO_2_ and N_2_ permeabilities of the FOTNIN/PTMSP MMM (4.64 × 10^4^ and 4.68 × 10^3^ Barrer, respectively) compared to those of the 17100N2/PTMSP MMM (4.30 × 10^4^ and 4.38 × 10^3^ Barrer, respectively).

For MOF/COF/PIM-1 MMMs, the percent changes in CO_2_ permeability, N_2_ permeability, and CO_2_/N_2_ selectivity are shown in Figure S3. The maximum increase in CO_2_ permeability was calculated as 6.63% when dual fillers (a MOF and a COF) were used instead of using only a COF filler in PIM-1. For all MOF/COF/PIM-1 MMMs, however, the percent change in N_2_ permeabilities was found to be relatively low (between −0.2 and 1.7%). Therefore, most of the MOF/COF/PIM-1 MMMs have a higher CO_2_/N_2_ selectivity compared to COF/polymer MMMs. Overall, our results showed that when a polymer with a relatively high gas permeability (≥10^4^ Barrer) like PTMSP is selected, CO_2_/N_2_ selectivities of the MOF/COF/polymer MMMs can be almost the same with or higher than the CO_2_/N_2_ selectivities of the COF/polymer MMMs. On the other hand, for a polymer with a mediocre gas permeability (∼10^3^ Barrer) like PIM-1, the number of COF/PIM-1 MMMs located below the upper bound is lower than the number of COF/PTMSP and COF/PTMSP-co(95/5) MMMs, and using a MOF as the second filler to generate MOF/COF/PIM-1 MMMs slightly improves their CO_2_ permeability and CO_2_/N_2_ selectivity.

### 3.2 CO_2_/CH_4_ Separation

**Figure 4:**
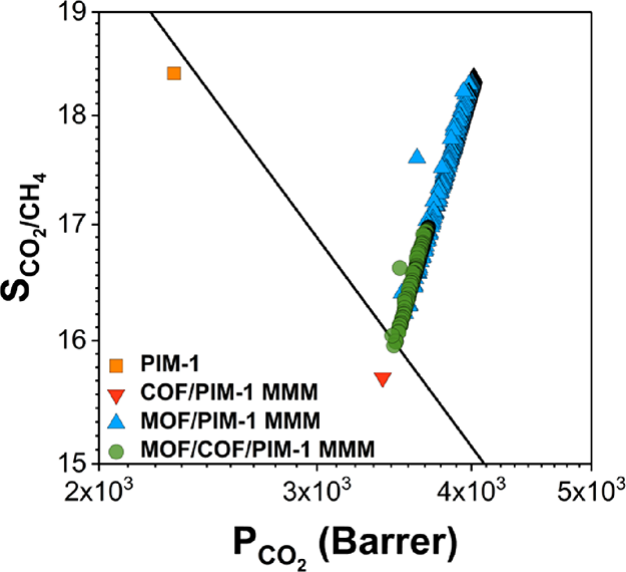
Calculated CO_2_ permeabilities and CO_2_/CH_4_ selectivities of 1143 MOF/PIM-1 MMMs, 21040N3/PIM-1 MMM, and 1143 MOF/COF/PIM-1 MMMs generated from them. Robeson’s upper bound is shown with the black line.

Almost all COF/PIM-1 MMMs among 589 COF/PIM-1 MMMs overcome the upper bound for CO_2_/CH_4_ separation except one (21040N3/PIM-1) as shown in Figure 4. When the COF filler, 21040N3, was used together with a MOF filler in PIM-1, 1143 out of 1193 MOF/21040N3/PIM-1 MMMs surpassed the upper bound. Figure 4 shows calculated CO_2_ permeabilities and CO_2_/CH_4_ selectivities of 1143 MOF/COF/PIM-1 MMMs (green data) generated from the combinations of 21040N3/PIM-1 MMM (red data) and 1143 MOF/PIM-1 MMMs (blue data). All the MOF/PIM-1 MMMs have a higher CO_2_ permeability (in the range of 3.51 × 10^3^–4.02 × 10^3^ Barrer) than the 21040N3/PIM-1 MMM (3.39 × 10^3^ Barrer). Similarly, CO_2_/CH_4_ selectivities of MOF/PIM-1 MMMs were calculated to be higher (16.2–18.4) than that of the 21040N3/PIM-1 MMM (15.7). Thus, the resulting MOF/COF/PIM-1 MMMs have a higher CO_2_ permeability, in the range of 3.45 × 10^3^–3.69 × 10^3^ Barrer, and a slightly higher CO_2_/CH_4_ selectivity, up to 17, compared to the 21040N3/PIM-1 MMM. The addition of MOF fillers to the 21040N3/PIM-1 MMM led to a percent increase in CO_2_ permeabilities between 1.8 and 8.9% and a percent increase in CO_2_/CH_4_ selectivities between 1.7 and 8.2% as shown in Figure S4. For example, when the 21040N3/PIM-1 MMM was combined with the CUFFEP/PIM-1 MMM, the MOF, CUFFEP, provided about an 8.8% increase in CO_2_ permeability. 21040N3 has low CO_2_ and CH_4_ permeabilities (1.71 × 10^4^ and 1.58 × 10^4^ Barrer, respectively), which can be attributed to slow diffusion of CO_2_ and CH_4_ (1.63 × 10^–6^ and 4.05 × 10^–6^ cm^2^/s, respectively) in the COF. On the other hand, fast CO_2_ diffusion (1.87 × 10^–4^ cm^2^/s) in the MOF led to a high CO_2_ permeability (1.57 × 10^6^ Barrer). As a result, the CUFFEP/21040N3/PIM-1 MMM overcomes the upper bound with a CO_2_ permeability of 3.69 × 10^3^ Barrer and a CO_2_/CH_4_ selectivity of 17.

As another example, the MOF FECKAA leads to the smallest percentage increase in CO_2_ permeability (1.8%) since the CO_2_ permeability of the FECKAA/PIM-1 MMM (3.51 × 10^3^ Barrer) is close to that of the 21040N3/PIM-1 MMM (3.39 × 10^3^ barrer). Still, even this slight increase in CO_2_ permeability with the use of FECKAA together with 21040N3 in PIM-1 is enough to locate the FECKAA/21040N3/PIM-1 MMM over the upper bound with a CO_2_ permeability of 3.45 × 10^3^ Barrer and a CO_2_/CH_4_ selectivity of 16. Overall, these results indicate that any COF can be incorporated into PIM-1 to obtain MMMs above the upper bound for CO_2_/CH_4_ separation if one of the 1143 MOFs is also used as the second filler in PIM-1.

### 3.3 H_2_/CH_4_, H_2_/N_2_, and H_2_/CO_2_ Separations

**Figure 5:**
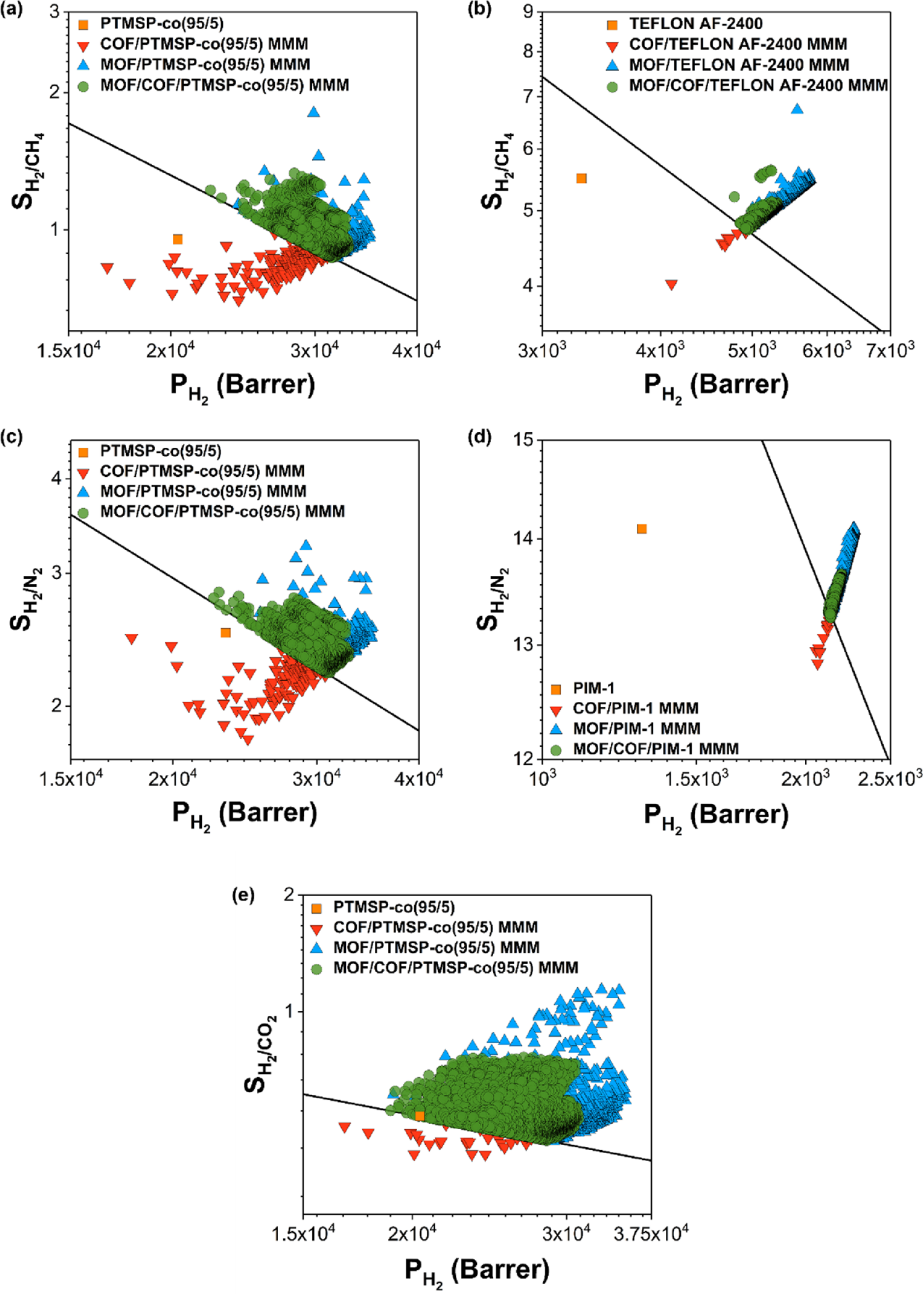
Calculated H_2_ permeability and H_2_/CH_4_ selectivity of (a) 574 MOF/PTMSP-co(95/5), 158 COF/PTMSP-co(95/5), and 40,904 MOF/COF/PTMSP-co(95/5) MMMs and (b) 1143 MOF/Teflon AF-2400, 8 COF/Teflon AF-2400, and 7830 MOF/COF/Teflon AF-2400 MMMs. Calculated H_2_ permeability and H_2_/N_2_ selectivity of (c) 653 MOF/PTMSP-co(95/5), 115 COF/PTMSP-co(95/5), and 41,603 MOF/COF/PTMSP-co(95/5) MMMs and (d) 1131 MOF/PIM-1, 9 COF/PIM-1, and 9735 MOF/COF/PIM-1 MMMs. Calculated H_2_ permeability and H_2_/CO_2_ selectivity of (e) 1018 MOF/PTMSP-co(95/5), 27 COF/PTMSP-co(95/5), and 22,090 MOF/COF/PTMSP-co(95/5) MMMs. Robeson’s upper bound is shown with the black line.

For H_2_/CH_4_ separation, we examined two different polymers, PTMSP-co(95/5) and Teflon AF-2400. PTMSP-co(95/5) is a highly permeable polymer with a relatively low selectivity (*P*_H_2__: 20,400 Barrer, *S*_H_2_/CH_4__: 0.953). We calculated H_2_ permeabilities and H_2_/CH_4_ selectivities of 189,687 MOF/COF/PTMSP-co(95/5) MMMs generated from 159 COF/PTMSP-co(95/5) MMMs and 1193 MOF/PTMSP-co(95/5) MMMs. MOF/PTMSP-co(95/5) MMMs have higher H_2_ permeabilities and much higher H_2_/CH_4_ selectivities (*P*_H_2__: 2.42 × 10^4^–3.50 × 10^4^ Barrer, *S*_H_2_/CH_4__: 0.9–1.8) than those of COF/PTMSP-co(95/5) MMMs (*P*_H_2__: 1.67 × 10^4^–3.11 × 10^4^ Barrer, *S*_H_2_/CH_4__: 0.7–1.0), which were used to generate MOF/COF/PTMSP-co(95/5) MMMs above the upper bound. As a result, as shown in Figure 5a, 40,904 MOF/COF/PTMSP-co(95/5) MMMs (green data) generated from 158 COF/PTMSP-co(95/5) MMMs (red data) and 574 MOF/PTMSP-co(95/5) MMMs (blue data) overcome the upper bound with H_2_ permeabilities and H_2_/CH_4_ selectivities in the ranges of 2.24 × 10^4^–3.29 × 10^4^ Barrer and 0.9–1.3, respectively. Changes in H_2_ and CH_4_ permeability and H_2_/CH_4_ selectivity of MMMs are presented in Figure S5. Many MOF/COF/PTMSP-co(95/5) MMMs exceed the upper bound thanks to the change in their *P*_H_2__ (up to 34%).

We then focused on Teflon AF-2400 to investigate the effects of using dual fillers in a relatively more H_2_-selective polymer (*P*_H_2__: 3300 Barrer, *S*_H_2_/CH_4__: 5.5). We calculated H_2_ permeabilities and H_2_/CH_4_ selectivities of 10,737 MOF/COF/Teflon AF-2400 MMMs generated from 9 COF/Teflon AF-2400 MMMs and 1193 MOF/Teflon AF-2400 MMMs. MOF/COF/Teflon AF-2400 MMMs (7830) (green data) generated from 8 COF/Teflon AF-2400 MMMs (red data) and 1143 MOF/Teflon AF-2400 MMMs (blue data) surpassed the Robeson’s upper bound as shown in Figure 5b. H_2_ permeabilities and H_2_/CH_4_ selectivities of MOF/Teflon AF-2400 MMMs (4.93 × 10^3^–5.76 × 10^3^ Barrer and 4.8–6.7, respectively) were found to be much higher than those of COF/Teflon AF-2400 MMMs (4.11 × 10^3^–4.92 × 10^3^ Barrer and 4.0–4.7, respectively).

As a result, incorporation of a MOF filler into Teflon AF-2400 together with a COF filler led to an increase in H_2_ permeability up to 16.5% and in H_2_/CH_4_ selectivity up to 29.3% as shown in Figure S6 compared to H_2_ permeability and H_2_/CH_4_ selectivity of COF/Teflon AF-2400 MMMs. H_2_ permeabilities of MOF/COF/Teflon AF-2400 MMMs exceeding the upper bound were found to be in the range of 4.79 × 10^3^–5.32 × 10^3^ Barrer, and their H_2_/CH_4_ selectivities varied between 4.7 and 5.6. These results suggest that, for dual filler-incorporated PTMSP-co(95/5) MMMs, the MOF–COF pair choice should be carefully made since the MOF can increase or decrease H_2_ and CH_4_ permeabilities of COF/PTMSP-co(95/5) MMMs. It is more straightforward to use different MOFs and COFs as fillers if the polymer matrix is Teflon AF-2400 since addition of the MOF into COF/Teflon AF-2400 MMMs generally improves the H_2_ permeability and H_2_/CH_4_ selectivities while does not significantly change CH_4_ permeabilities.

For H_2_/N_2_ separation, we focused on two polymers, PTMSP-co(95/5) and PIM-1, and calculated H_2_ permeabilities and H_2_/N_2_ selectivities of MOF/COF/PTMSP-co(95/5) and MOF/COF/PIM-1 MMMs. A total of 143,160 MOF/COF/PTMSP-co(95/5) MMMs were studied for H_2_/N_2_ separation. MOF/PTMSP-co(95/5) MMMs (653) (blue data) and 115 COF/PTMSP-co(95/5) MMMs (red data) have H_2_ permeabilities in the range of 2.56 × 10^4^–3.5 × 10^4^ and 1.78 × 10^4^–3.07 × 10^4^ Barrer, respectively. These wide ranges of H_2_ permeabilities led to 41,603 types of MOF/COF/PTMSP-co(95/5) MMMs (green data in Figure 5c) surpassing the upper bound with high H_2_ permeabilities (2.24 × 10^4^–3.28 × 10^4^ Barrer). Figure S7 demonstrates that H_2_ permeabilities and H_2_/N_2_ selectivities of dual filler-incorporated MMMs were improved up to 39.1 and 32.6% compared to those of COF/PTMSP-co(95/5) MMMs, respectively, which means that the MOF filler plays a significant role in determining the H_2_ permeability and H_2_/N_2_ selectivity of the MOF/COF/PTMSP-co(95/5) MMMs.

PIM-1 has a moderate H_2_/N_2_ selectivity (14.1) and H_2_ permeability (1300 barrer). When PIM-1 is used as the matrix of MMMs, 9735 MOF/COF/PIM-1 MMMs (green data in Figure 5d) generated from 9 COF/PIM-1 MMMs (red data) and 1131 MOF/PIM-1 MMMs (blue data) overcome Robeson’s upper bound among a total of 13,123 MOF/COF/PIM-1 MMMs. H_2_ permeabilities of 1131 MOF/PIM-1 MMMs and 9 COF/PIM-1 MMMs vary between 2.14 × 10^3^ and 2.27 × 10^3^ and 2.05 × 10^3^ and 2.13 × 10^3^ Barrer, respectively, and their H_2_/N_2_ selectivities were found to vary between 13.3−14.1, and 12.8−13.2, respectively, as shown in Figure 5d. As a result, 9735 MOF/COF/PIM-1 MMMs above the upper bound have H_2_ permeability and H_2_/N_2_ selectivity in the range of 2.12 × 10^3^–2.20 × 10^3^ Barrer and 13.3–13.7, respectively. As Figure S8 shows, addition of the MOF filler increased the H_2_ permeability and H_2_/N_2_ selectivity of MOF/COF/PIM-1 MMMs up to 5.2 and 4.8%.

Finally, for H_2_/CO_2_ separation, H_2_ permeabilities and H_2_/CO_2_ selectivities were calculated for a total of 32,211 MOF/COF/PTMSP-co(95/5) MMMs generated from 27 COF/PTMSP-co(95/5) MMMs below the upper bound and 1193 MOF/PTMSP-co(95/5) MMMs. One thousand eighteen of 1193 MOF fillers improved the H_2_/CO_2_ separation performances of all 27 COF-incorporated PTMSP-co(95/5) membranes. MOF/COF/PTMSP-co(95/5) MMMs (22,090) surpassed the upper bound for H_2_/CO_2_ separation with H_2_ permeabilities of 1.89 × 10^4^–3.09 × 10^4^ Barrer as shown with green data in Figure 5e. The percent increases in H_2_ permeabilities of dual filler-incorporated PTMSP-co(95/5) membranes compared to those of only COF-incorporated MMMs reached up to 45% as shown in Figure S9. Similar to H_2_/CH_4_ separation, it is required to choose a suitable MOF–COF pair to incorporate into PTMSP-co(95/5) since the MOF filler can cause an undesirable decrease in H_2_ and CO_2_ permeabilities of the MMMs.

**Figure 6:**
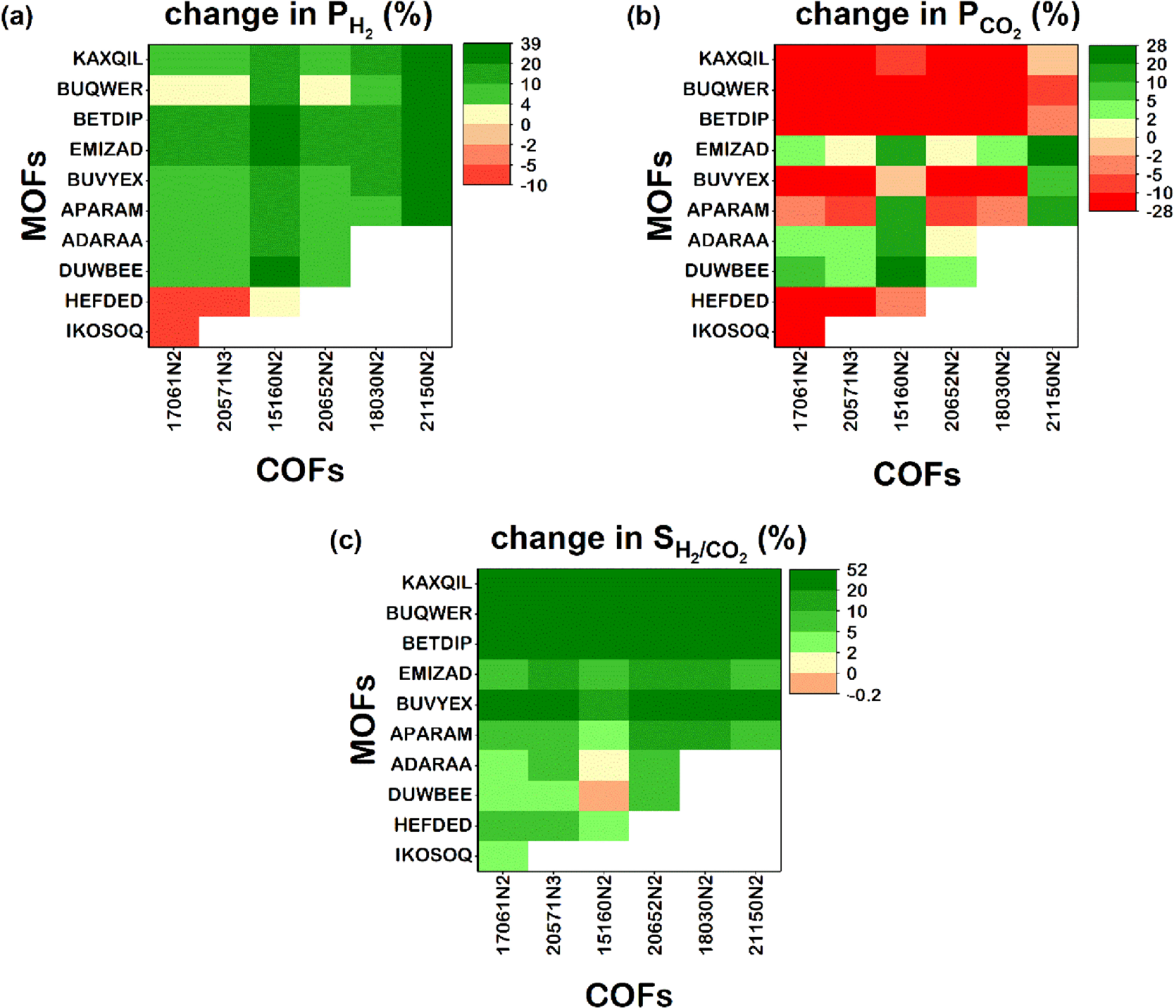
Calculated percent changes in (a) H_2_ permeabilities, (b) CO_2_ permeabilities, and (c) H_2_/CO_2_ selectivities of representative MOF/COF/PTMSP-co(95/5) MMMs with respect to COF/PTMSP-co(95/5) MMMs.

The percent changes in H_2_ permeability, CO_2_ permeability, and H_2_/CO_2_ selectivity for a representative set of MOF/COF/PTMSP-co(95/5) MMMs are shown in Figure 6. For example, when the MOF, KAXQIL, and the COF, 15160N2, were used together in PTMSP-co(95/5), both H_2_ permeability and H_2_/CO_2_ selectivity of the MMM increased by 17 and 27%, respectively, compared to those of only COF-incorporated MMMs. A higher self-diffusivity of H_2_ in the MOF, KAXQIL (1.08 × 10^–3^ cm^2^/s), compared to that in the COF, 15160N2 (5.30 × 10^–4^ cm^2^/s), led to a much higher H_2_ permeability of the MOF (1.46 × 10^5^ Barrer) compared to the COF (2.84 × 10^4^ Barrer). Another promising MOF–COF pair is ADARAA and 15160N2, which led to a 20% higher H_2_ and CO_2_ permeability compared to using only 15160N2 in PTMSP-co(95/5). As a result, the ADARAA/15160N2/PTMSP-co(95/5) MMM overcomes the upper bound. Overall, these results show the great promise of using two types of dual fillers in polymer membranes for H_2_ separation.

To understand how the identity of MOF fillers affects the gas separation performances of dual filler-incorporated polymer membranes, we specifically focused on two MOF–COF pairs, KAXQIL-17061N2 and IKOSOQ-17061N2 with the same COF, 17061N2. The KAXQIL/PTMSP-co(95/5) MMM has a slightly higher H_2_ permeability (2.99 × 10^4^ Barrer) than the 17061N2/PTMSP-co(95/5) MMM (2.70 × 10^4^ Barrer). This was attributed to the high H_2_ permeability of KAXQIL (1.46 × 10^5^ Barrer) due to high H_2_ diffusivity (1.08 × 10^–3^ cm^2^/s) in this MOF. On the other hand, the IKOSOQ/PTMSP-co(95/5) MMM exhibited a lower H_2_ permeability (2.24 × 10^4^ Barrer) than the 17061N2/PTMSP-co(95/5) MMM since the COF filler is more H_2_-permeable (7.86 × 10^4^ Barrer) than the MOF, IKOSOQ (3.19 × 10^4^ Barrer). Therefore, depending on the MOF–COF pair, an MOF/COF/polymer can exceed the upper bound as a result of an increase in permeability and selectivity together or due to an increase in selectivity only.

To explain why MOF/COF/polymer MMMs exhibit higher gas permeabilities with respect to those of only COF-incorporated MMMs, we examined the structural and chemical properties of MOFs and COFs. Gas permeability is a result of complex interplay of several physical and chemical properties, and it is difficult to isolate the impact of each property on the gas permeability. We aimed to focus on structural properties mainly driving the materials’ affinity toward a gas species and focused on the top MOFs, which led to the highest percent increase in CO_2_ permeabilities of MOF/COF/PTMSP MMMs compared to COF/PTMSP MMMs for CO_2_/N_2_ separation. Table S4 presents the structural properties of the top 15 MOF fillers that led to the highest increase in CO_2_ permeabilities when utilized in MOF/COF/PTMSP MMMs. These top 15 MOF fillers were found to have some common structural properties such as moderate PLDs (between 4.28 and 11.7 Å), high porosities (0.66–0.75), relatively high LCDs (5.87–12.16 Å), and large *S*_acc_ (2229.2–4307.1 m^2^/g). These relatively high porosities and LCD values lead to high CO_2_ permeabilities of MOFs. On the other hand, the COF used in these dual filler-incorporated polymer membranes has larger PLD and LCD values (9.49 and 9.92 Å, respectively), a smaller accessible surface area (607.2 m^2^/g), and a smaller porosity (0.48) compared to the top 15 MOF fillers. We also examined the organic units of each framework; except an MOF, CORZIU, all the top 15 MOFs have benzene-based ligands, and 5 of the top MOFs have amine-based ligands in agreement with the previous reports showing that amine-based ligands in frameworks provide high CO_2_ affinities,^40,41^ which generally lead to high CO_2_ permeability. The most common metals in MOFs that we studied are zinc (Zn), found in 260 MOFs, copper (Cu) in 230 MOFs, cadmium (Cd) in 127 MOFs, and cobalt (Co) in 126 MOFs, as shown in Figure S10. Four of the top MOFs have Zn and Cd metals, and 3 of them include nickel (Ni). High CO_2_ uptakes of 11 MOFs, which have Zn and Cd, can be attributed to the stronger interaction between CO_2_ molecules and these metals (energetic interaction values, ε/*k*_B_, for these metals are 62.4 and 114.8 K, respectively). Although Ni has a relatively low ε/k_B_ value (7.6 K), three MOFs with Ni have a very large surface areas (>3600 m^2^/g), and they also include amine-based ligands and fluorine. All these factors resulted in high CO_2_ uptakes and hence high CO_2_ permeabilities for these MOFs.

Table S5 shows PLD, LCD, *S*_acc_, and porosity of the top 15 MOF fillers, which led to the highest increase in H_2_ permeabilities of MOF/COF/PTMSP-co(95/5) MMMs, and the COF fillers used to generate these MMMs for H_2_/CH_4_, H_2_/N_2_, and H_2_/CO_2_ separations. PLD, LCD, and porosities of the top MOFs are generally larger (9.24–34.51 Å, 18.21–37.53 Å, and 0.64–0.93, respectively) than those of COFs used to generate the dual filler-incorporated membranes (4.86–13.23 Å, 6.31–13.64 Å, and 0.62–0.63, respectively). Smaller PLDs, LCDs, and porosities of COFs lead to low H_2_ permeabilities. When these MOFs and COFs were incorporated into PTMSP-co(95/5), the generated MOF/COF/PTMSP-co(95/5) MMMs show higher H_2_ permeabilities compared to COF/PTMSP-co(95/5) MMMs. Thirteen out of the 15 MOFs have amine-based ligands. Among the top 15 MOFs, Cu was found in 5 MOFs, and Zn was found in 3 MOFs. Among these top 15 MOFs, there are MOFs with Zn and indium (In) (ε/*k*_B_ values for LJ interactions are 62.4 and 301.6 K, respectively), which lead to relatively high H_2_ uptakes and large porosities that lead to H_2_ diffusivities, providing high H_2_ permeabilities. On the other hand, COFs listed in Table S5 offer lower H_2_ uptakes and diffusivities and hence lower H_2_ permeabilities than the top MOFs.

In this work, we computed gas separation performances of almost a million types of MOF/COF/polymer MMMs, and our computations have several assumptions that need to be discussed. These assumptions may not be valid in real applications, but they save enormous computational time when a million of MMMs has been studied. We assumed rigid MOFs and COFs in molecular simulations and modeled MMMs as defect-free structures. The Maxwell permeation model, which assumes an ideal morphology without considering voids, filler agglomeration, and polymer rigidification in the MMM, was used to predict the gas permeabilities of MMMs. It should be noted that fillers in MMMs can cause an increased rigidity at the polymer–filler interface, which indicates a good compatibility and adhesion at the organic–inorganic interfaces between fillers and polymers.^42^ The morphologies of fillers and filler agglomeration, which depends on many intrinsic factors such as the degree of dispersion of particles and the surface area of particles, can affect the gas separation performances of MMMs.^43,44^ Our simulations do not provide any information about these issues.

There are also recent examples of core-and-shell MOF@COF (a MOF confined in the pore of a COF) composites incorporated into a polymer for gas separation applications. A UiO-66-NH_2_@TpPa-1 composite was recently incorporated into polysulfone (PSF) to be utilized as a filler in an MMM for the separation of an equimolar CO_2_/CH_4_ mixture at 1 bar and 298 K.^21^ Due to the high compatibility of the COF in the outer layer with the polymer matrix, nonselective void formation between the filler and the polymer was avoided, which led to an increase in CO_2_/CH_4_ selectivity and CO_2_ permeability. Since our work focused on dual filler-incorporated MOF/COF/polymer membranes instead of MMMs including MOF@COF composites, we did not compare the performances of MOF/COF/polymer MMMs studied in this work with the performances of MOF@COF incorporated polymer MMMs that recently appeared in the literature.

## 4 Conclusions

In this work, by combining molecular simulations with theoretical permeation models, we predicted CO_2_ capture and H_2_ purification performances of the largest number of membranes, 966,330 MOF/COF/polymer MMMs studied to date. All permeability data were obtained at 1 bar and 298 K. Many MOF/COF/polymer MMMs were shown to exceed the upper bounds established for polymers. For H_2_ and CO_2_ separations, the majority of COF/polymer MMMs below the upper bound overcome the upper bound when an MOF was used as a second type of filler in the MOF/COF/polymer MMM. The CO_2_ permeabilities of MOF/COF/PTMSP MMMs and MOF/COF/PIM-1 MMMs were computed to vary between 4.10 × 10^4^ and 4.72 × 10^4^ and 3.68 × 10^3^ and 3.82 × 10^3^ barrer, respectively. These values were significantly higher than the CO_2_ permeabilities of neat PTMSP (2.9 × 10^4^ barrer) and PIM-1 (2.3 × 10^3^ barrer) membranes. For CO_2_/CH_4_ separation, dual filler-incorporated MOF/COF/PIM-1 MMMs were found to have a higher CO_2_ permeability, in the range of 3.45 × 10^3^–3.69 × 10^3^ barrer, and a slightly higher CO_2_/CH_4_ selectivity, up to 17, compared to only COF-incorporated PIM-1 membranes. H_2_ permeabilities of MOF/COF/PTMSP-co(95/5) MMMs were calculated to range from 1.89 × 10^4^ to 3.09 × 10^4^ barrer, which are higher than the permeability of a neat polymer membrane, 2.04 × 10^4^ barrer. H_2_ permeability (selectivity) of Teflon for H_2_/CH_4_ and PIM-1 for H_2_/N_2_ separations were 3.3 × 10^3^ Barrer (5.5) and 1.3 × 10^3^ Barrer (14.1), respectively, whereas those values for MOF/COF/Teflon and MOF/COF/PIM-1 MMMs vary between 4.79 × 10^3^ and 5.32 × 10^3^ Barrer (4.7−5.6) and 2.14 × 10^3^ and 2.20 × 10^3^ Barrer (13.3−13.7). Overall, we examined if there is a significant potential in utilizing dual types of fillers in MMMs to achieve higher gas permeabilities and/or selectivities compared to those offered by the MMMs having a single type of filler. We utilized the enormous data for MOF/polymer MMMs and COF/polymer MMMs that we have produced for the selected polymers having either high gas permeabilities or high gas selectivities. The polymers that we focused on were selected based on the permeability- selectivity limit defined by the Robeson’s upper bound introduced in 2008, which is still widely used in high-throughput computational screening studies to compare the gas separation performances of MMMs with that of pure polymers. Therefore, we compared the performances of MOF/COF/polymer MMMs with respect to this upper bound. In future works, the performance of MOF/COF/polymer MMMs should be also investigated for polymers having high permeabilities together with high selectivities, and should be compared with respect to the updated upper bounds.^45,46^ It is not trivial to draw an overall conclusion valid for all MOF/COF/polymers since there are cases that using dual fillers in a polymer provides significant improvements in gas separation performance for some MOF/COF/polymer combinations, while for others, it does not make a significant change. However, our results demonstrated that a significant portion of MOF/COF/polymer MMMs surpass the permeability-selectivity limit when dual fillers are used in a polymer instead of merely a COF filler. Therefore, we believe that our results will highlight the potential of MOF/COF/polymer MMMs and accelerate the design and development of novel MOF/COF/polymer MMMs for various gas separations.2 × 10^3^ and 2.20 × 10^3^ barrer (13.3–13.7). Overall, the aim of this work is to examine if there is a significant potential in utilizing dual types of fillers in MMMs to achieve higher gas permeabilities and/or selectivities compared to those offered by the MMMs having a single type of filler. For this aim, we utilized the enormous data for MOF/polymer MMMs and COF/polymer MMMs that we have produced for the selected polymers having either high gas permeabilities or high gas selectivities. The polymers that we focused on were selected based on the permeability–selectivity limit defined by the Robeson’s upper bound introduced in 2008, which is still widely used in high-throughput computational screening studies to compare the gas separation performances of MMMs with that of pure polymers. Therefore, we compared the performances of MOF/COF/polymer MMMs with respect to this upper bound. In future works, the performance of MOF/COF/polymer MMMs should be also investigated with polymers, which have high permeabilities together with high selectivities, and should be compared with respect to the updated upper bounds.^45,46^ It is not trivial to draw an overall conclusion valid for all MOF/COF/polymers since there are cases that using dual fillers in a polymer provides significant improvements in gas separation performance for some of the MOF/COF/polymer combinations, while for others, it does not make a significant change. However, our results demonstrated that a significant portion of MOF/COF/polymer MMMs surpass the permeability–selectivity limit when dual fillers are used in a polymer instead of merely a COF filler. Therefore, we believe that our results will highlight the potential of MOF/COF/polymer MMMs and accelerate the design and development of novel MOF/COF/polymer MMMs for various gas separations.

## Supporting Information Available

Details for GCMC and MD simulations of COFs and MOFs; experimental gas permeabilities and membrane selectivities of neat polymers for CO_2_/N_2_, CO_2_/CH_4_, H_2_/CH_4_, H_2_/N_2_, and H_2_/CO_2_ separations; number of all COF/polymer MMMs, which are below the upper bound and total combinations of MOF/COF/polymer MMMs; comparison of experimental and computed gas permeabilities of MOF/COF/polymer MMMs; percent changes in gas permeabilities and selectivities of MOF/COF/polymer MMMs compared to COF/polymer MMMs for different gas separations; structural properties of the top 15 MOFs as second fillers in MOF/COF/PTMSP MMMs and MOF/COF/PMTSP-co(95/5) MMMs for CO_2_ separation and H_2_ purification, respectively (PDF)
