# Supplementary material for: Computational Investigation of Dual Filler-Incorporated Polymer Membranes for Efficient CO2 and H2 Separation: MOF/COF/Polymer Mixed Matrix Membranes
Source: Ind Eng Chem Res. 2023 Jan 26;62(6):2924–36. doi: 10.1021/acs.iecr.2c04500 (PMC9936544; doi:10.1021/acs.iecr.2c04500)
Supplement: Supplementary file 1 — ie2c04500_si_001.pdf [file ie2c04500_si_001.pdf]

Supporting Information

Computational Investigation of Dual Filler-Incorporated Polymer Membranes for Efficient CO_2 and H_2 Separation: MOF/COF/polymer Mixed Matrix Membranes

Sena Aydin[a], Cigdem Altintas[b], Ilknur Erucar[c], Seda Keskin[b*]

[a] Department of Computational Science and Engineering, Koc University, Rumelifeneri Yolu, Sariyer, 34450, Istanbul, Turkey

[b] Department of Chemical and Biological Engineering, Koc University, Rumelifeneri Yolu, Sariyer, 34450, Istanbul, Turkey

[c] Department of Natural and Mathematical Sciences, Ozyegin University, Cekmekoy, 34794, Istanbul, Turkey

Submitted to *Industrial & Engineering Chemistry Research*

*Corresponding author. Email: skeskin@ku.edu.tr Phone: +90(212)338 1362

Table S1. Parameters and conditions of GCMC and MD simulations for COFs and MOFs.

**Parameters/Conditions**	**COFs**	**MOFs**
**GCMC**		
Number of cycles	20000	50000
Number of initialization cycles	10000	20000
External temperature (K)	298	298
External pressure (bar)	1	1
Cut-off radius (Å)	14	13
**MD**		
Ensemble type	NVT	NVT
Time step	1 fs (total 5 ns)	1 fs (total 5 ns)
Thermostat	Nose-Hoover	Nose-Hoover
External temperature (K)	298	298

Table S2. Experimental gas permeabilities and membrane selectivities of polymers.

**CO_2/N_2**			
	$P^{P}_{CO_2}$ (Barrer)	$S^{P}_{CO_2/N_2}$	Ref.
PTMSP	29,000	10.7	1
PIM-1	2300	25	2
**CO_2/CH_4**			
	$P^{P}_{CO_2}$ (Barrer)	$S^{P}_{CO_2/CH_4}$	Ref.
PIM-1	2300	18.4	2
**H_2/CH_4**			
	$P^{P}_{H_2}$ (Barrer)	$S^{P}_{H_2/CH_4}$	Ref.
PTMSP-co (95/5)	20,400	0.953	3
Teflon AF-2400	3300	5.5	4
**H_2/N_2**			
	$P^{P}_{H_2}$ (Barrer)	$S^{P}_{H_2/N_2}$	Ref.
PTMSP-co (95/5)	20,400	2.5	3
PIM-1	1300	14.1	2
**H_2/CO_2**			
	$P^{P}_{H_2}$ (Barrer)	$S^{P}_{H_2/CO_2}$	Ref.
PTMSP-co (95/5)	20,400	0.538	3

Table S3. Number of all COF/polymer MMMs which were found to be located below the upper bound, all MOF/polymer MMMs, and total combinations of MOF/COF/polymer MMMs.

**Polymer**	**MOF/COF/polymer MMM**	**COF/polymer MMM**	**MOF/polymer MMM**
		**CO_2/N_2**	
PTMSP	572,640	480	1193
PIM-1	3579	3	1193
		**CO_2/CH_4**	
PIM-1	1193	1	1193
		**H_2/CH_4**	
PTMSP-co(95/5)	189,687	159	1193
TEFLON AF-2400	10,737	9	1193
		**H_2/N_2**	
PTMSP-co(95/5)	143,160	120	1193
PIM-1	13,123	11	1193
		**H_2/CO_2**	
PTMSP-co(95/5)	32,211	27	1193

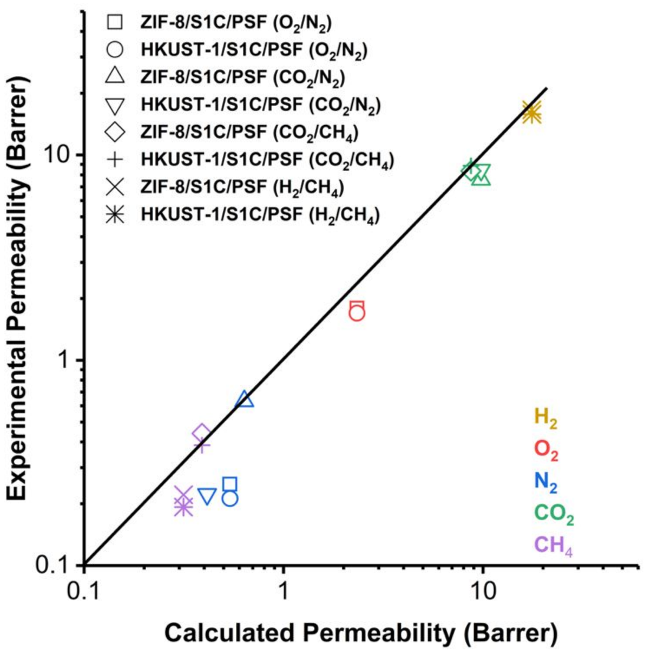

Figure S1. Comparison of experimental and computed gas permeabilities of dual filler-incorporated polymer membranes following the approach presented in this study. Yellow, red, blue, green, and violet colors represent H_2, O_2, N_2, CO_2, and CH_4 permeabilities of dual filler-incorporated polymer membranes at 2.75 bar, 308 K. The mixtures of corresponding gases are indicated in parenthesis. Gas permeabilities of MOFs were calculated in this work while the data for S1C and PSF were taken from the literature.[5]

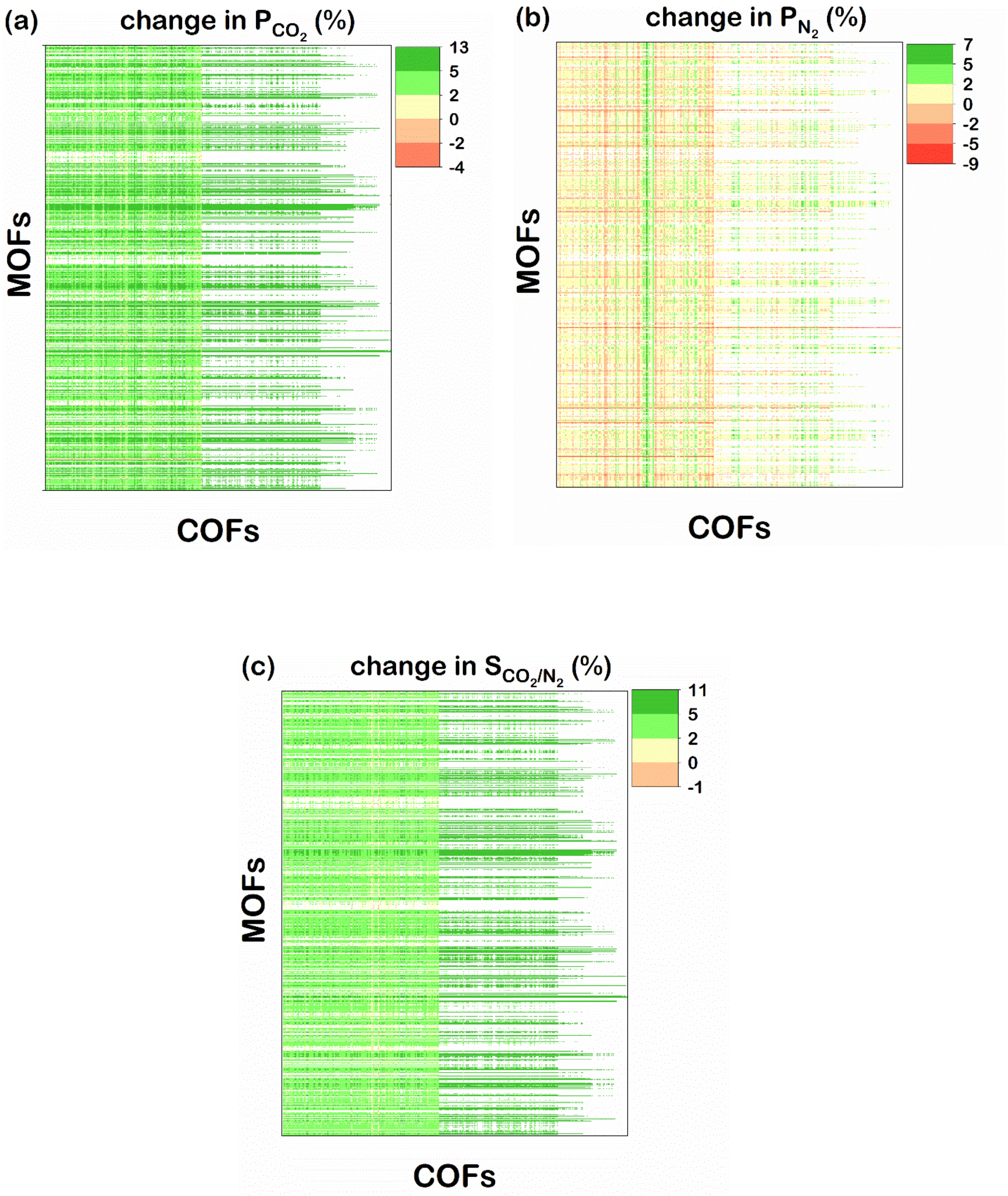

Figure S2. Calculated percent change in (a) CO_2 permeabilities, (b) N_2 permeabilities, (c) CO_2/N_2 selectivities of 119,622 MOF/COF/PTMSP MMMs compared to those of 375 COF/PTMSP MMMs.

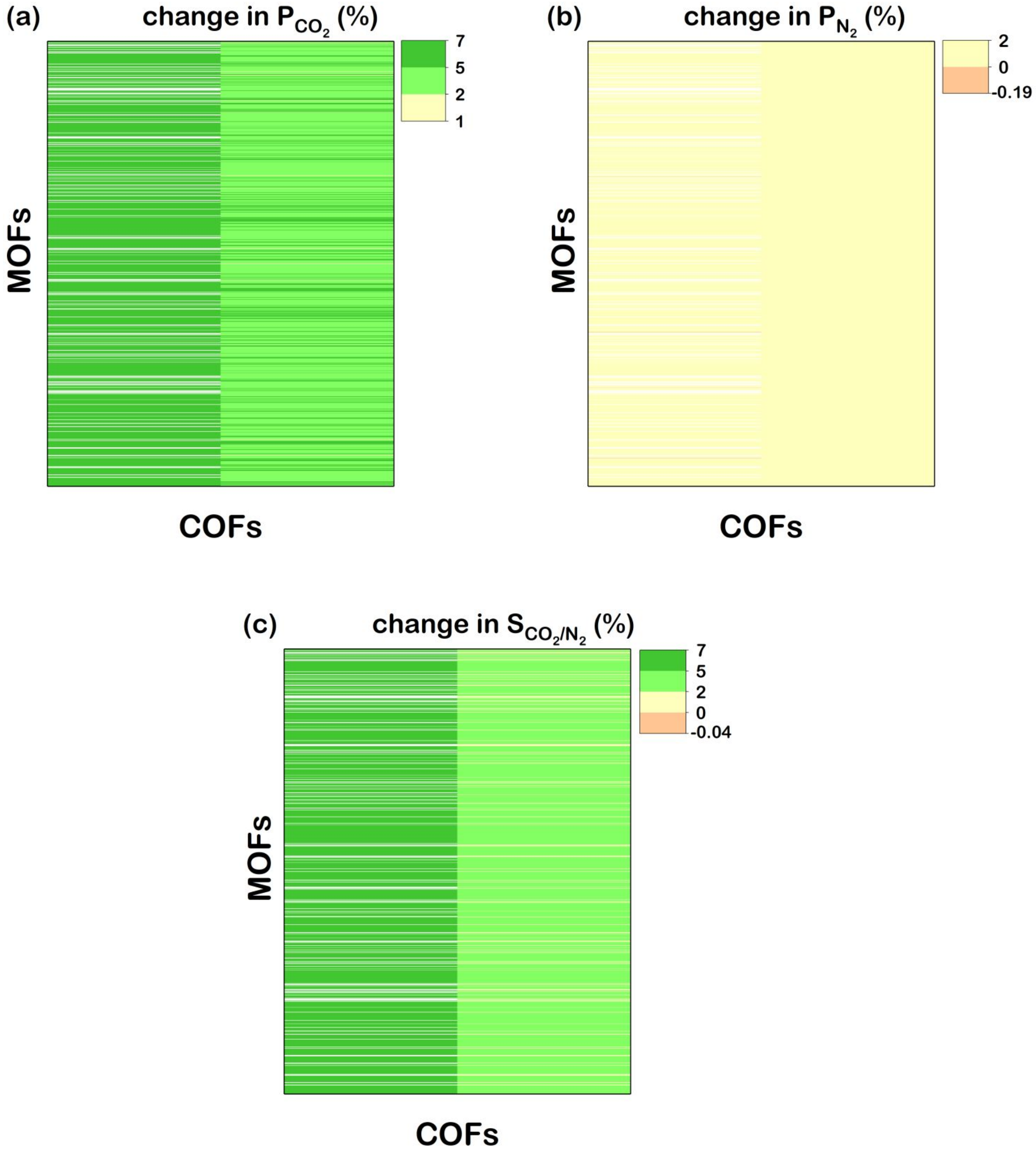

Figure S3. Calculated percent change in (a) CO_2 permeabilities, (b) N_2 permeabilities, (c) CO_2/N_2 selectivities of 2020 MOF/COF/PIM-1 MMMs compared to those of 2 COF/PIM-1 MMMs.

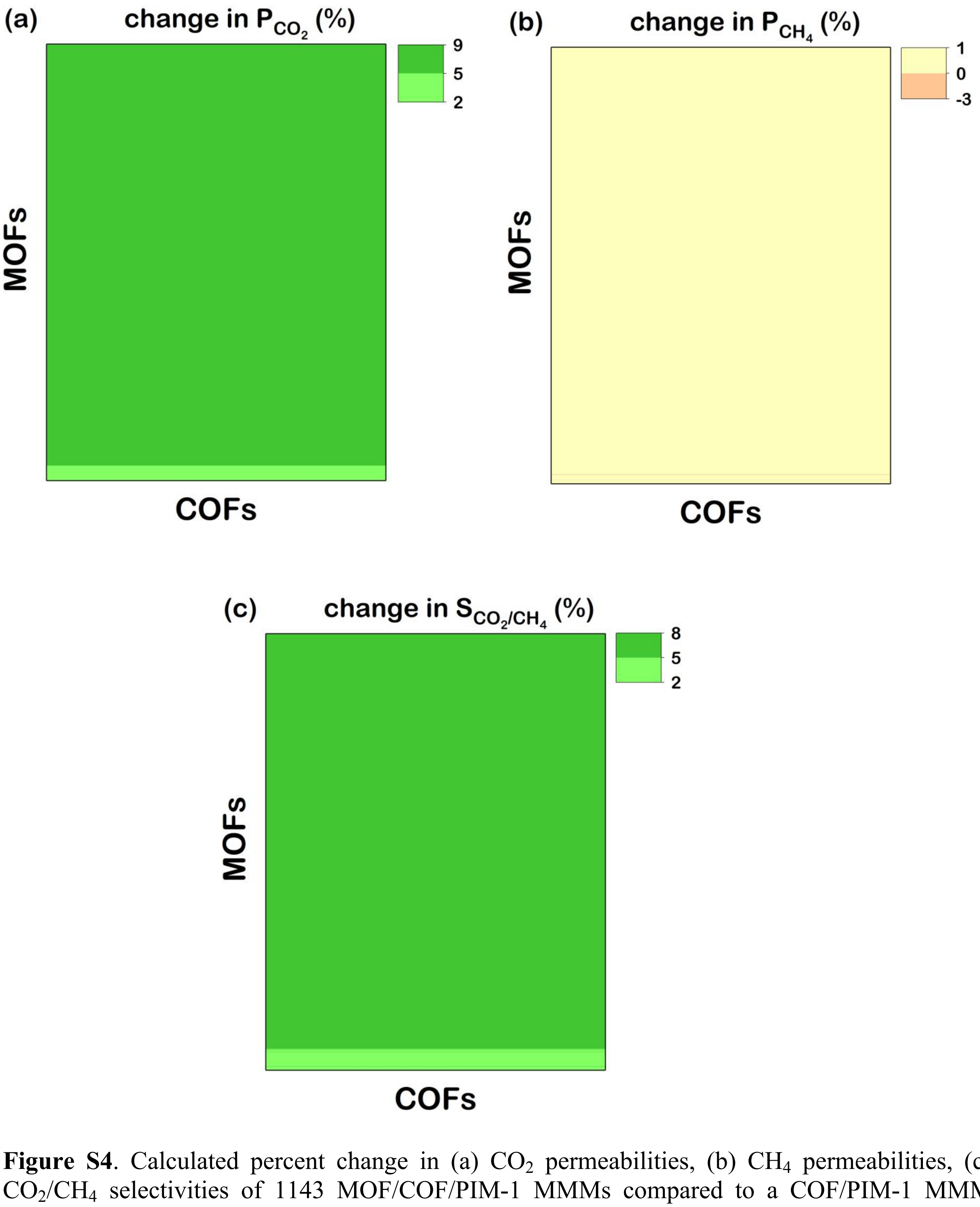

Figure S4. Calculated percent change in (a) CO_2 permeabilities, (b) CH_4 permeabilities, (c) CO_2/CH_4 selectivities of 1143 MOF/COF/PIM-1 MMMs compared to a COF/PIM-1 MMM (21040N3/PIM-1).

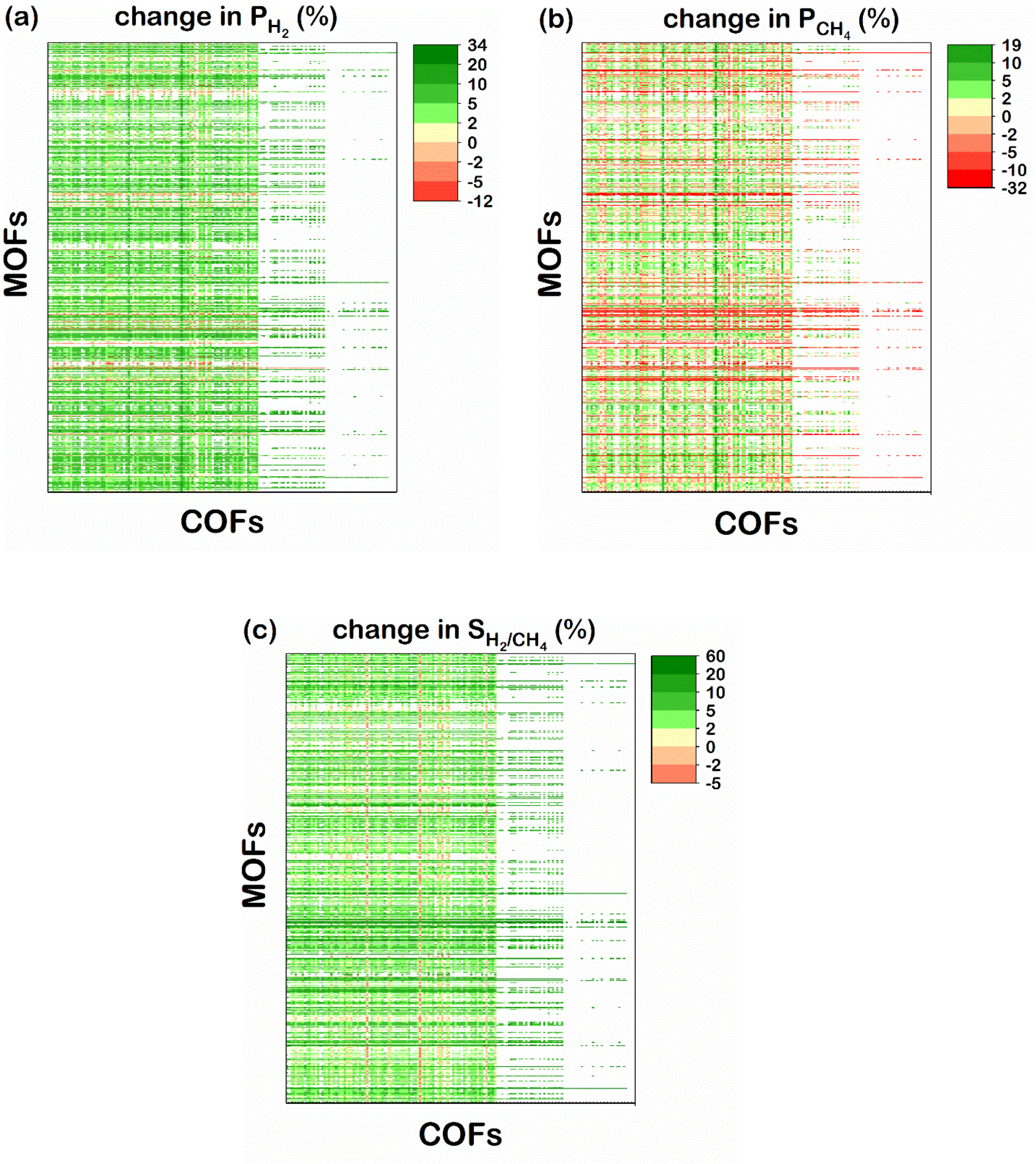

Figure S5. Calculated percent change in (a) H_2 permeabilities, (b) CH_4 permeabilities, (c) H_2/CH_4 selectivities of 40,904 MOF/COF/PTMSP-co(95/5) MMMs compared to those of 158 COF/PTMSP-co(95/5) MMMs.

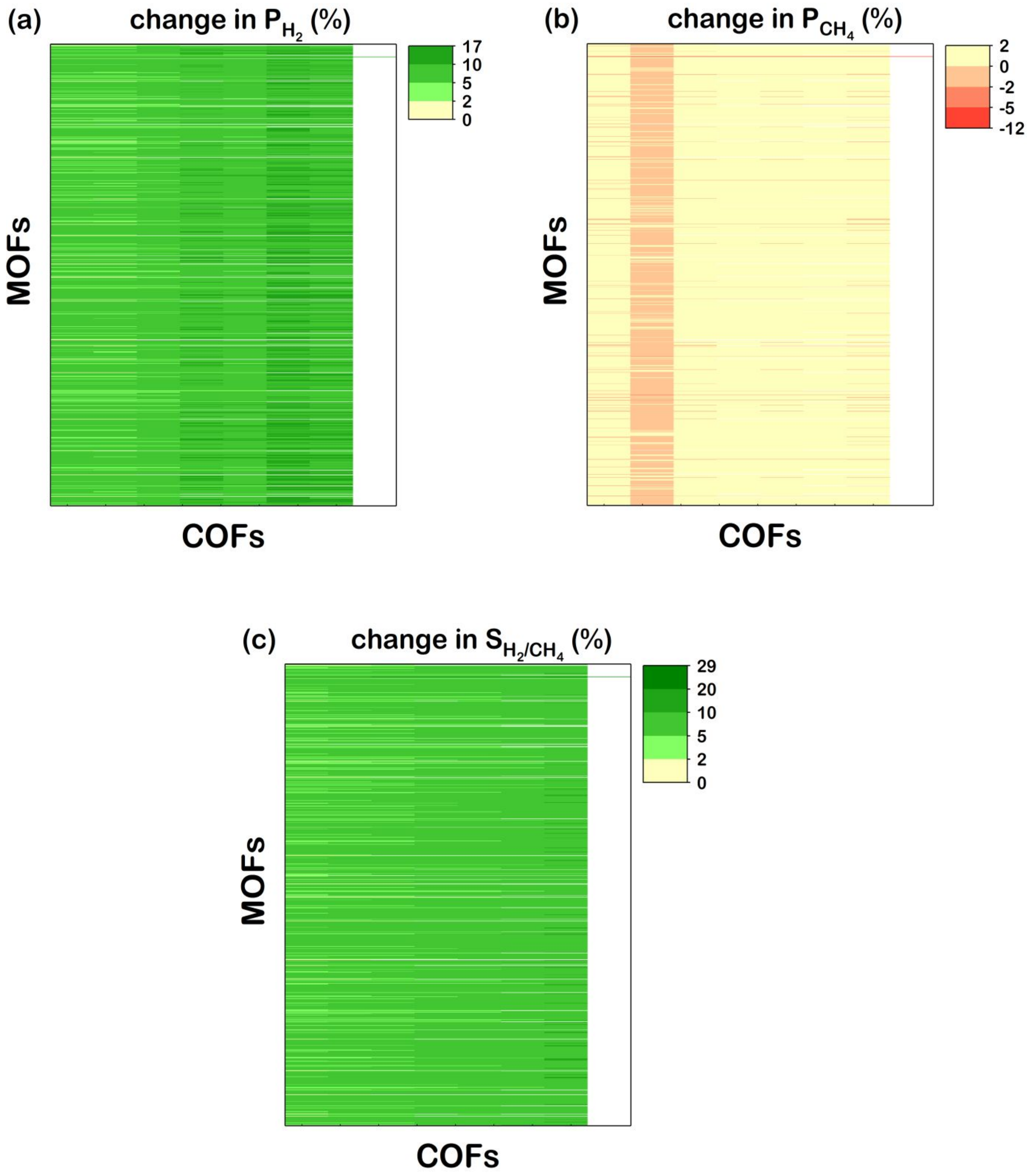

Figure S6. Calculated percent change in (a) H_2 permeabilities, (b) CH_4 permeabilities, (c) H_2/CH_4 selectivities of 7830 MOF/COF/Teflon AF-2400 MMMs compared to those of 8 COF/Teflon AF-2400 MMMs.

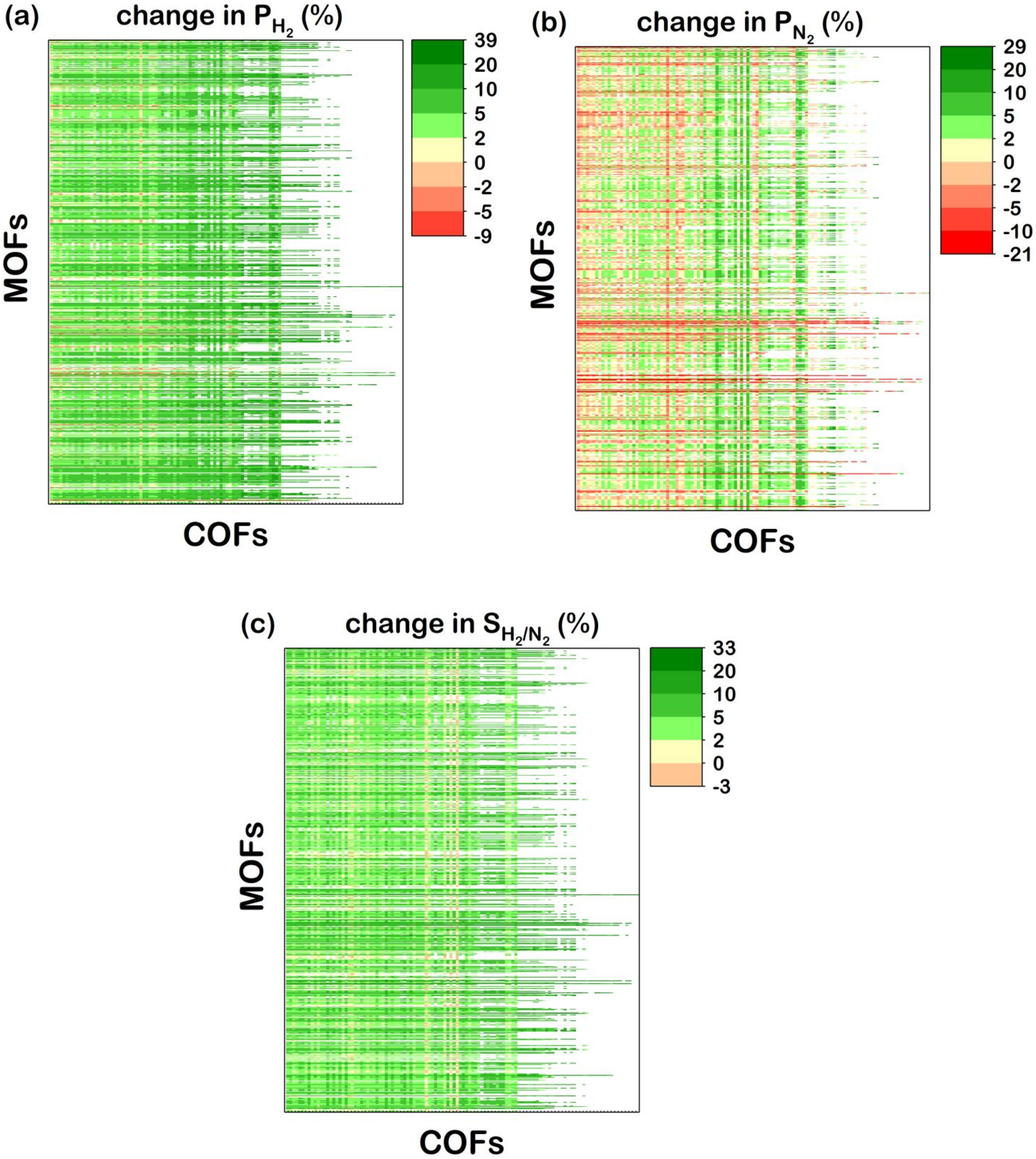

Figure S7. calculated percent change in (a) H_2 permeabilities, (b) N_2 permeabilities, (c) H_2/N_2 selectivities of 41,603 MOF/COF/PTMSP-co(95/5) MMMs compared to those of 115 COF/PTMSP-co(95/5) MMMs.

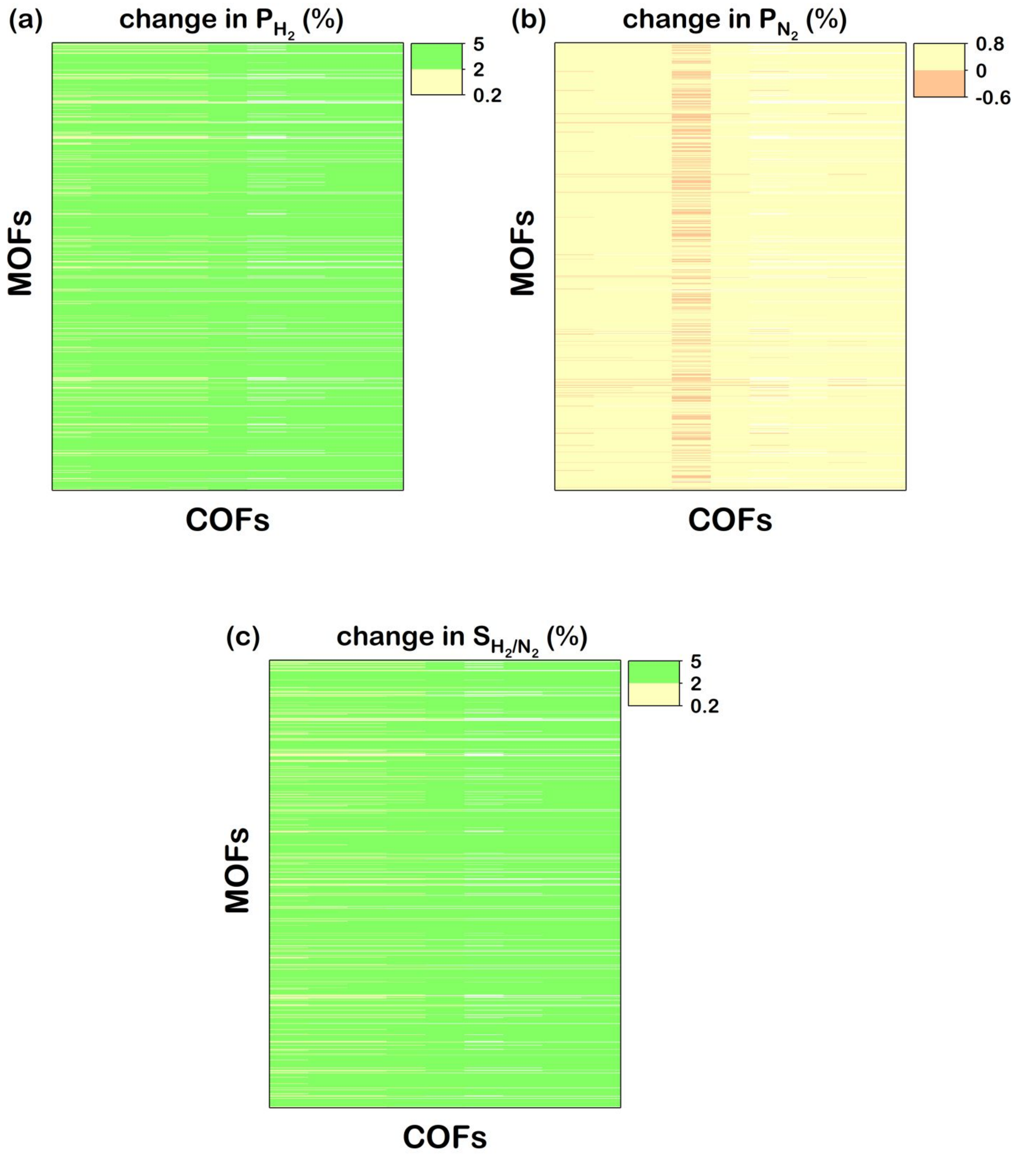

Figure S8. Calculated percent change in (a) H_2 permeabilities, (b) N_2 permeabilities, (c) H_2/N_2 selectivities of 9735 MOF/COF/PIM-1 MMMs compared to those of 9 COF/PIM-1 MMMs.

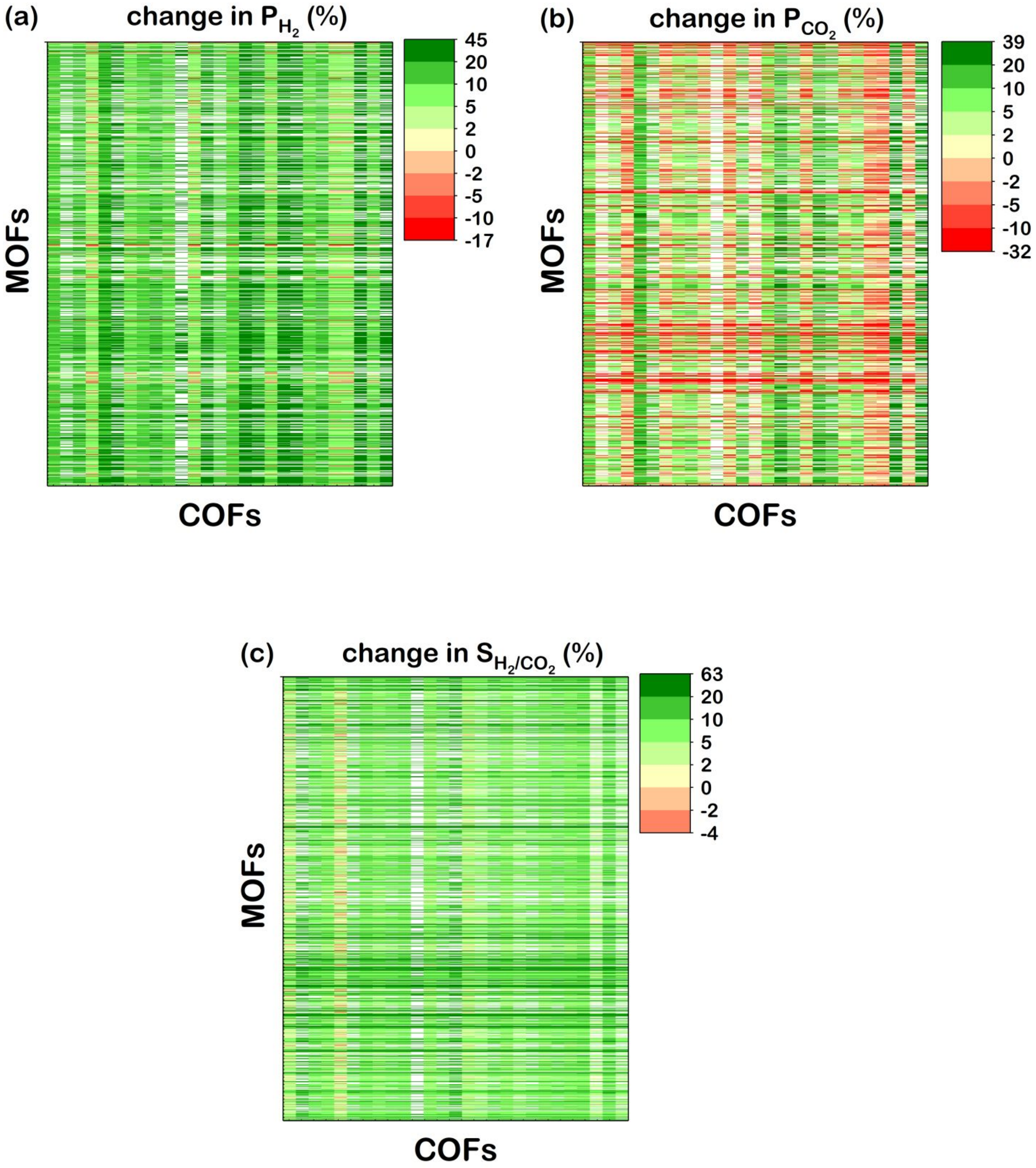

Figure S9. Calculated percent change in (a) H_2 permeabilities, (b) CO_2 permeabilities, (c) H_2/CO_2 selectivities of 22,090 MOF/COF/PTMSP-co(95/5) MMMs compared to those of 27 COF/PTMSP-co(95/5) MMMs.

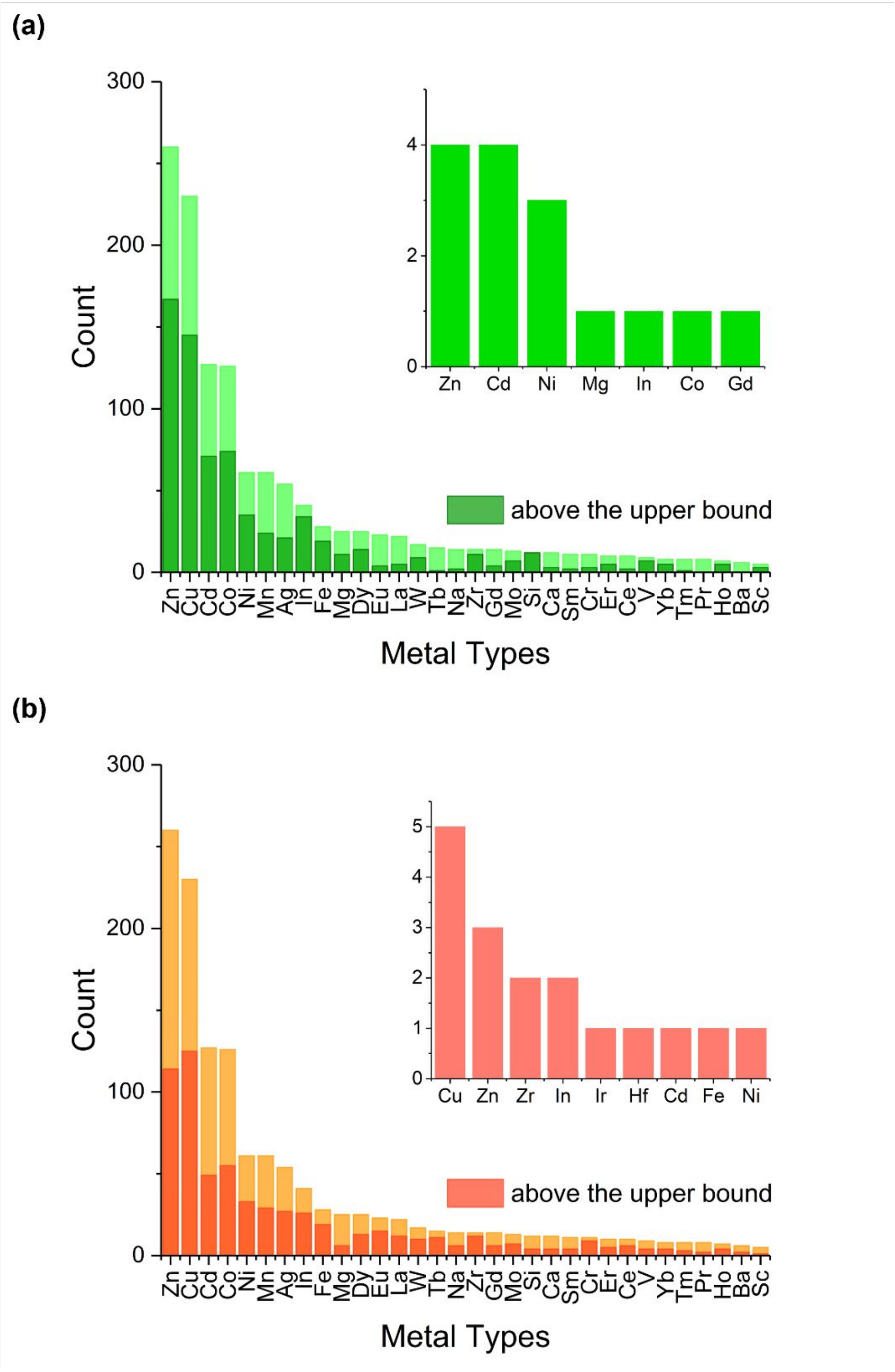

Figure S10. Distributions of metal types in MOFs used to generate (a) MOF/COF/PTMSP MMMs for CO_2/N_2, (b) MOF/COF/PTMSP-co(95/5) MMMs for H_2/CH_4 separations. Inner plots show the distributions of metal types in the top 15 MOFs used to generate MOF/COF/polymer MMMs that are above the upper bound for each separation when the dual filler-incorporated membranes were sorted in terms of a percent increase in CO_2 and H_2 permeabilities with respect to COF/polymer MMMs.

Table S4. Structural properties of the top 15 different MOFs and their COF pair(s) which led to the highest percent increase in CO_2 permeabilities of MOF/COF/PTMSP MMMs compared to COF/PTMSP MMMs for CO_2/N_2 separation.

Name	PLD (Å)	LCD (Å)	Surface Area (m^2/g)	Porosity
DUZPUL	4.92	7.07	2751.0	0.69
COQTEJ	6.63	7.76	3807.5	0.70
FIPJAR01	5.63	6.16	2604.4	0.75
DUWBEE	6.03	7.47	2466.2	0.72
AVEQID	6.23	7.55	2819.8	0.70
CORZIU	11.70	12.16	2988.0	0.75
FUDHAP	6.52	7.29	3112.4	0.71
BEXPAX	5.83	6.18	4307.1	0.71
AVESOL	4.28	5.87	2500.0	0.66
FIRNUR	6.04	6.62	3654.0	0.69
FIRNIF	6.06	6.79	3613.7	0.69
DURDIF	6.05	6.94	3594.1	0.72
GACBOE	4.36	6.67	2229.2	0.67
DUDKUK	6.66	8.85	2880.0	0.75
ATEYOP	8.17	10.53	3319.2	0.74
COF: 16260N2	9.49	9.92	607.2	0.48

Table S5. Structural properties of the top 15 different MOFs and their COF pair(s) which led to the highest percent increase in H_2 permeabilities of MOF/COF/PTMSP-co(95/5) MMMs compared to COF/PTMSP-co(95/5) MMMs for H_2/CH_4, H_2/N_2, and H_2/CO_2 separations.

Name	PLD (Å)	LCD (Å)	Surface Area (m^2/g)	Porosity
FOTNIN	29.93	33.62	3183.7	0.92
AVAJUE	19.54	37.53	6088.9	0.93
DEYNIG	3.77	4.67	161.2	0.42
ADUROI	17.12	25.69	3194.1	0.84
KAWHEY	15.59	18.21	3256.2	0.81
ALEJAE	14.57	19.87	5195.2	0.89
AVILEY	10.77	25.43	2452.6	0.84
GUPBEZ02	10.85	23.87	1789.6	0.80
FUWXOL	4.43	4.77	831.5	0.52
KEJBOQ	3.93	5.18	792.3	0.54
EMIZAD	34.51	35.59	3737.0	0.85
AWUPAL	9.24	18.42	5001.1	0.85
HOHMEX	14.89	18.78	4996.4	0.88
IZERAI	21.12	21.82	2170.7	0.78
CUNFOH01	5.77	8.65	1059.2	0.64
COF: 11001N2	4.86	6.31	1627.8	0.63
COF: 16190N2	6.85	8.78	2061.5	0.62
COF: 09010N2	13.23	13.64	1679.2	0.63

References

(1) Mizumoto, T.; Masuda, T.; Higashimura, T. Polymerization of [o-(trimethylgermyl)phenyl] acetylene and polymer characterization. *J. Polym. Sci., Part A: Polym. Chem.* **1993**, *31* (10), 2555-2561.
(2) Budd, P. M.; Msayib, K. J.; Tattershall, C. E.; Ghanem, B. S.; Reynolds, K. J.; McKeown, N. B.; Fritsch, D. Gas separation membranes from polymers of intrinsic microporosity. *J. Membr. Sci.* **2005**, *251* (1), 263-269.
(3) Nagai, K.; Higuchi, A.; Nakagawa, T. Gas permeability and stability of poly(1-trimethylsilyl-1-propyne-co-1-phenyl-1-propyne) membranes. *J. Polym. Sci., Part B: Polym. Phys.* **1995**, *33* (2), 289-298.
(4) Pinnau, I.; Toy, L. G. Gas and vapor transport properties of amorphous perfluorinated copolymer membranes based on 2,2-bistrifluoromethyl-4,5-difluoro-1,3-dioxole/tetrafluoroethylene. *J. Membr. Sci.* **1996**, *109* (1), 125-133.
(5) Zornoza, B.; Seoane, B.; Zamaro, J. M.; Téllez, C.; Coronas, J. Combination of MOFs and zeolites for mixed-matrix membranes. *ChemPhysChem* **2011**, *12* (15), 2781-2785.
